# Rodent-Related Zoonotic Pathogens at the Human–Animal–Environment Interface in Qatar: A Systematic Review and Meta-Analysis

**DOI:** 10.3390/ijerph18115928

**Published:** 2021-05-31

**Authors:** Md Mazharul Islam, Elmoubashar Farag, Ahmad Mahmoudi, Mohammad Mahmudul Hassan, Ehsan Mostafavi, Khalid A. Enan, Hamad Al-Romaihi, Muzzamil Atta, Abdel Rahim M. El Hussein, Zilungile Mkhize-Kwitshana

**Affiliations:** 1 Department of Animal Resources, Ministry of Municipality and Environment, Doha P.O. Box 35081, Qatar; khalid.enan@gmail.com (K.A.E.), maabdalla@mme.gov.qa (M.A.); 2 School of Laboratory Medicine and Medical Sciences, College of Health Sciences, University of KwaZulu Natal, Durban 4000, South Africa; 3 Ministry of Public Health, Doha P.O. Box 42, Qatar; halromaihi@moph.gov.qa; 4 Department of Biology, Faculty of Science, Urmia University, Urmia 5756151818, Iran; a.mahmoudi@urmia.ac.ir; 5 Faculty of Veterinary Medicine, Chattogram Veterinary and Animal Sciences University, Chattogram 4225, Bangladesh; miladhasan@yahoo.com; 6 Department of Epidemiology and Biostatistics, Research Centre for Emerging and Reemerging Infectious Diseases, Pasteur Institute of Iran, Tehran 1316943551, Iran; mostafaviehsan@gmail.com; 7 National Reference Laboratory for Plague, Tularemia and Q Fever, Research Centre for Emerging and Reemerging Infectious Diseases, Pasteur Institute of Iran, Akanlu, Kabudar Ahang, Hamadan 6556153145, Iran; 8 Department of Virology, Central Laboratory, The Ministry of Higher Education and Scientific Research, Khartum 7099, Sudan; abdelhussein@hotmail.com; 9 College of Animal Production, Bahri University, Khartoum 11111, Sudan; 10 School of Life Sciences, College of Agriculture, Engineering & Science, University of KwaZulu Natal, Durban 4000, South Africa; mkhizekwitshanaz@ukzn.ac.za; 11 South African Medical Research Council, Cape Town 7505, South Africa

**Keywords:** pathogens, rodents, public health, environment, meta-analysis, One Health, Qatar

## Abstract

Rodents are one of the most diversified terrestrial mammals, and they perform several beneficial activities in nature. These animals are also important as carriers of many pathogens with public health importance. The current systematic review was conducted to formulate a true depiction of rodent-related zoonoses in Qatar. Following systematic searches on PubMed, Scopus, Science Direct, and Web of Science and a screening process, a total of 94 published articles were selected and studied. The studied articles reported 23 rodent-related zoonotic pathogens that include nine bacterial, eleven parasitic, and three viral pathogens, from which the frequently reported pathogens were *Mycobacterium tuberculosis* (32 reports), *Escherichia coli* (23), and *Salmonella* spp. (16). The possible pathway of entry of the rodent-borne pathogens can be the land port, seaports, and airport of Qatar through carrier humans and animals, contaminated food, and agricultural products. The pathogens can be conserved internally by rodents, pets, and livestock; by agricultural production systems; and by food marketing chains. The overall estimated pooled prevalence of the pathogens among the human population was 4.27% (95%CI: 4.03–4.51%; *p* < 0.001) with significant heterogeneity (*I*^2^ = 99.50%). The top three highest prevalent pathogens were *M.* *tuberculosis* (30.90%; 22.75–39.04%; *p* < 0.001; *I*^2^ = 99.70%) followed by *Toxoplasma gondii* (21.93%; 6.23–37.61%; *p* < 0.001; *I*^2^ = 99.30%) and hepatitis E virus (18.29%; 11.72–24.86%; *p* < 0.001; *I*^2^ = 96.70%). However, there is a knowledge gap about the listed pathogens regarding the occurrence, transmission pathways, and rodent role in transmission dynamics at the human–animal–environment interface in Qatar. Further studies are required to explore the role of rodents in spreading zoonotic pathogens through the One Health framework, consisting of zoologists, ecologists, microbiologists, entomologists, veterinarians, and public health experts in this country.

## 1. Introduction

Rodentia is one of the most diversified mammalian orders in the world [1]. With 2552 known species, they make up 39.3% of mammals and are the essential components of many terrestrial ecosystems. These animals have several beneficial activities in nature, such as soil aeration and insect control [2,3,4]. However, rodents are also sources of zoonotic pathogens [4,5,6]. Almost 10% of the global rodent population are either carriers or reservoirs of pathogens with public health importance [5,6]. Rodents transfer infectious agents to humans by direct contact with humans and animals or through contamination of human or animal food and water with rodent stool, hair, and urine. Arthropod vectors on rodent skin are also able to carry several zoonotic pathogens [5,6,7,8]. Rodent-borne diseases and their prevalence are associated with several factors, including the rodent population, human socio-economic lifestyle, human conflict, and war [7,9,10,11]. Human-related activities such as migration, large-scale traveling, trade, urbanization, and agricultural activities can also be facilitating factors in transferring rodent-borne pathogens from one community to another [12,13].

Qatar is a small desert country located on the coast of the Arabian Peninsula [14]. The country is inhabited by multinational people from around 94 countries around the world [15]. The current population of the country is 2.8 million [16], of whom only 10.5% are Qatari nationals. The people who make up around 80% of the Qatar population are mainly from India, Bangladesh, Nepal, Egypt, the Philippines, Pakistan, Sri Lanka, Sudan, Syria, Jordan, Lebanon, Kenya, and Iran [15]. Approximately 83% of these non-Qatari residents are primarily construction workers, housemaids, drivers, and retail market workers [17]. As a desert country, agriculture is limited [18], and the country imports live animal and food products from nearby countries such as Iran, Turkey, India, Pakistan, and Bangladesh [19,20].

The rodent fauna of the country is limited to four species, which include three commensal species (*Mus musculus, Rattus norvegicus,* and *Rattus rattus*) and a single wild rodent species (*Jaculus loftusi*, previously known as *Jaculus jaculus*) [21,22,23,24]. *M. musculus, R. norvegicus*, and *R. rattus* are reported to spread rodent-borne zoonoses among the human population throughout the world [8,25]. The countries from where most of the Qatari residents originated and some of the countries from where food and agricultural products are imported are endemic with several rodent-borne diseases, including leishmaniasis, enteric fever, echinococcosis, and hepatitis E virus [26,27,28]. For effective prevention and control measures of such diseases, it is essential to know the status of these pathogens in Qatar. However, to the authors’ knowledge, no studies have been performed to understand the rodent-borne diseases in this country at the human–animal–environment interface. Therefore, the current study aimed to identify the rodent-related zoonotic pathogens detected in humans, animals, and environmental sources in Qatar and possible transmission pathways and to estimate the pooled prevalence of these pathogens among humans in this country.

## 2. Materials and Methods

**Figure 1:**
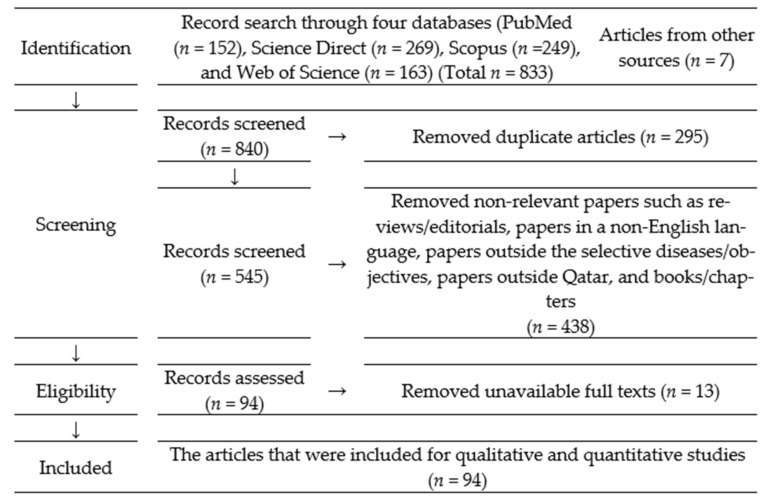
PRISMA flow diagram describing selection of published articles on rodent-related diseases with public health importance in Qatar and the inclusion/exclusion process used in the study.

We conducted a systematic review following the Preferred Reporting Items for Systematic Reviews and Meta-Analysis (PRISMA) guidelines [29]: (1) We conducted a database search to find relevant articles, (2) we assessed the relevance of the searched articles, and (3) we extracted data from the included articles (Figure 1, and Supplementary Table S1).

### 2.1. Data Search

In the beginning, we conducted a mini-review to determine the list of rodent-borne diseases. We found a total of 88 diseases that can have public health importance (Supplementary Table S2). Among these, 26 were bacterial diseases, 2 fungal, 27 parasitic, and 33 viral diseases. Then, we conducted a systematic literature search from 20 to 26 March 2020 through four databases: PubMed, Scopus, Science Direct, and Web of Science. The search included all the original field reports for each of the 88 rodent-borne diseases individually in Qatar with no time limit of publication. The search terms included ((disease name OR synonym OR causal agent[s]) AND Qatar). We screened the searches as “Title/Abstract” in PubMed, “Find articles with these terms” in Science Direct, “TITLE-ABS-KEY” in Scopus, and “Topic” in Web of Science.

### 2.2. Assessing the Searched Articles

We compiled the searched document on the EndNote X9 system (Clarivate Analytics, Philadelph, PA, USA). Using EndNote X9, we identified and removed the duplicate articles. After that, two authors assessed the title and abstract of the articles. The articles that had unknown relevance based on the title and abstract study were subjected to full-text screening. We included only original research studies published in English. We excluded articles that did not fulfill the objective, were reviews/editorials, were outside the selective diseases, were from outside Qatar, or were books/chapters.

### 2.3. Data Extraction

The extracted data included the study type and season, the pathogen, target population, total sample tested and total positives, and associating factors of a disease prevalence and dynamics (Supplementary Table S3).

### 2.4. Data Analysis

We collected the relevant data in a Microsoft Excel spreadsheet and analyzed them using the statistical software STATA/IC-13.0 (Stata Corp, 4905 Lakeway Drive, College Station, TX 77845, USA). Descriptive statistics of the selected articles were calculated and expressed as percentage (%) and 95% confidence interval (CI). Then, the crude prevalence estimation was calculated by dividing the total number of individual positive pathogens with the total number of sampled and expressed percentages (%). The crude estimate of prevalence was used for the 95% confidence interval (CI), the *p*-value, and heterogeneity (*I^2^*). A random-effect meta-analysis model was applied using the “mean” command specifying random due to the study’s high degree of heterogeneity(*I^2^* > 80%) [30]. The output was illustrated using a forest plot.

## 3. Results

### 3.1. Characteristics of the Studied Articles

The literature search resulted in a total of 94 articles published from 1991 to 2020 (Table 1). Many of the articles (*n* = 42, 44.68%, 95%CI: 34.41–55.29%) were published by last five years (2016–2020), and only one (*n* = 1, 1.06%; 0.027–5.79%) was published between 1991–1995. The studies were mostly conducted in human hosts (*n* = 80, 85.11%, 95%CI: 76.28–91.61), followed by animals (*n* = 10, 10.64%; 5.22–18.70), and the environment (*n* = 1, 1.06%, 95%CI: 0.027–5.79), with some studies on the human–animal–environment interface. The majority of the studies assessed rodent-related bacteria (*n* = 62, 65.96%, 95%CI: 55.46–75.42), followed by helminths (*n* = 10, 10.64%, 95%CI: 5.22–18.70), protozoa (*n* = 9, 9.57%; 95%CI: 4.47–17.40), and viruses (*n* = 5, 5.32%, 95%CI: 1.75–11.98). However, some articles described mixed infections.

### 3.2. Possible Transmission Pathways of the Pathogens in Qatar

**Figure 2:**
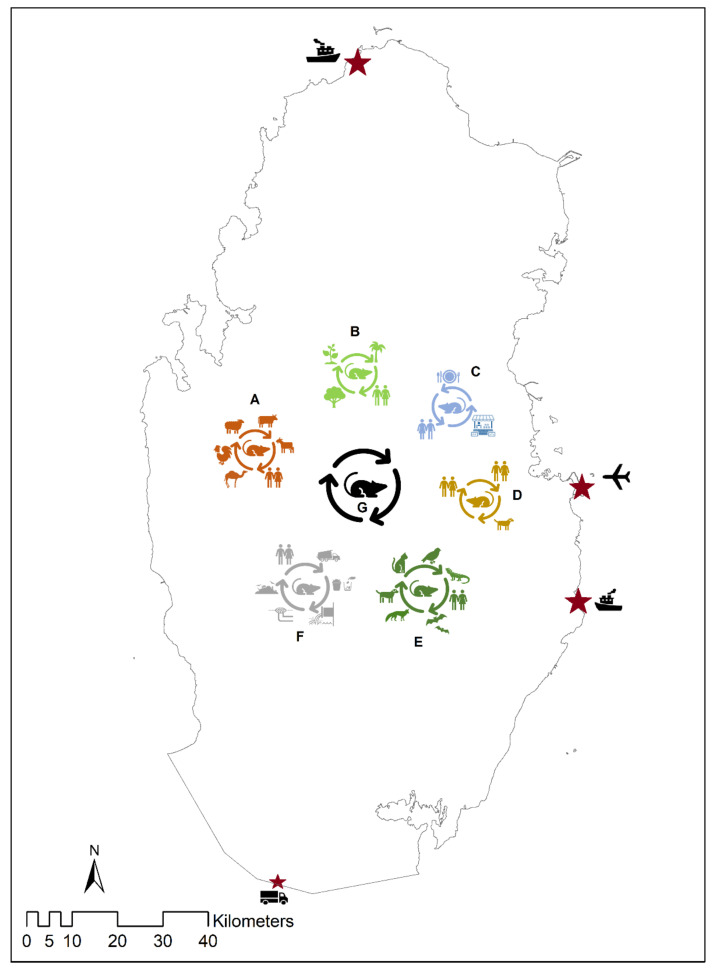
Possible transmission pathways of the rodent-related zoonotic pathogens at the human–animal–environmental interface in Qatar. The stars indicate the plausible routes of entry of rodent-related pathogens in Qatar via carrier immigrants and the importing of contaminated food and agricultural products. “A” indicates that rodents can be a source of transmission of pathogens among livestock animals and humans inside Qatar. Similarly, the figure illustrates how rodents can facilitate zoonotic pathogens transmission among agricultural products and humans “B”, residential areas between humans and pet animal “C”, in the environment between stray cats and dogs, wildlife, and humans “D”, fresh food in households, restaurants, markets, and humans “E”, and through the sewage system “F”. Rodents can interlink zoonotic pathogens “G” between A, B, C, D, E, and F.

The current review shows that besides humans, rodent-related zoonotic pathogens are available among livestock, stray (free on-street) and domesticated cats and dogs, big cats (cheetah), and environmental samples. In addition, rodents are usually available in every facility of an ecosystem, such as animal farms, agricultural farms, residential areas, desert ecosystems, restaurants, and sewage facilities in Qatar. Therefore, rodents can contribute to zoonotic pathogen transmission within and between these facilities. The possible transmission pathways of rodent-borne zoonotic pathogens in Qatar are illustrated in Figure 2. Moreover, the land port at the Qatar–Saudi Arabia border, the international airport, and the two seaports can also contribute to rodent-associated zoonoses transmission into Qatar by the human migration and transmission of live animals, rodents, and agricultural products from different parts of the world.

### 3.3. Estimated Pooled Prevalence of Pathogens

**Figure 3:**
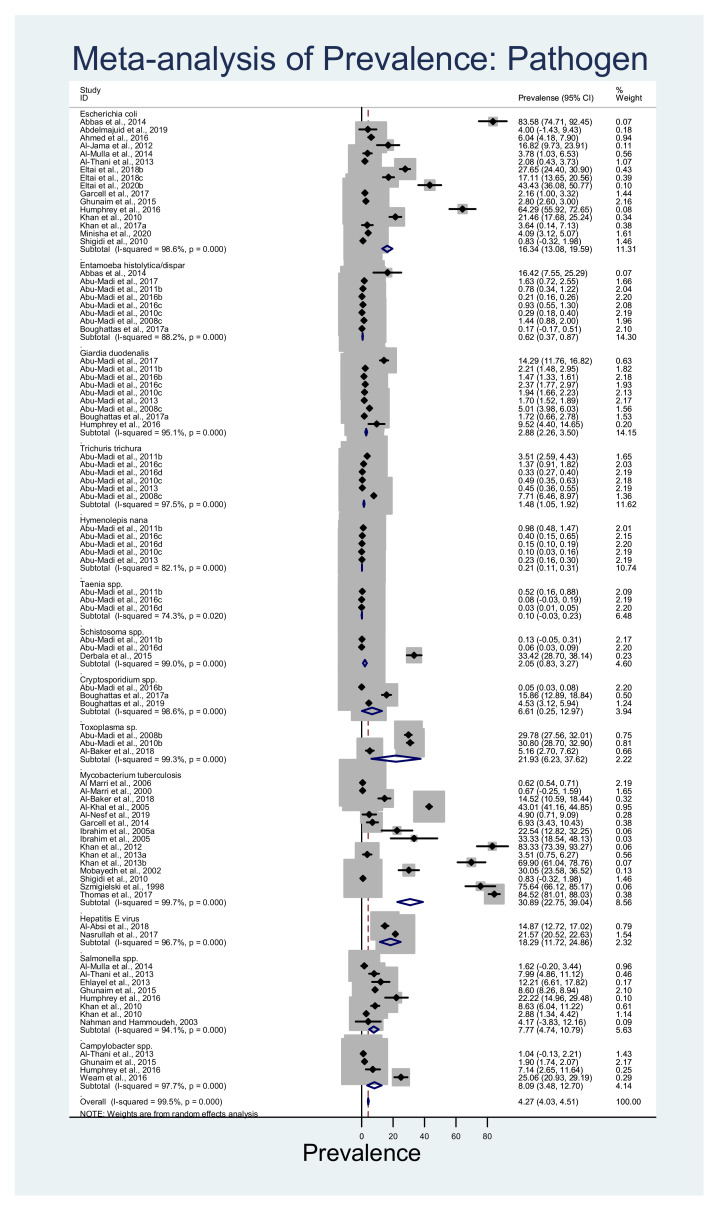
Forest plot of the pooled overall prevalence of rodent-related pathogens in Qatar. The central square represents point estimates, whereas the square size represents the weight of each study in the meta-analysis.

The overall estimated pooled prevalence of the rodent-related zoonotic pathogens within the human population in Qatar was 4.27% (95%CI: 4.03–4.51%; *p* < 0.001) with significant heterogeneity (*I*^2^ = 99.50%) and *p*-value (*p* = 0.00) (Figure 3). Among the individual pathogens, the estimated pooled prevalence of *Mycobacterium tuberculosis* was the highest (30.90%; 22.75–39.04%; *p* < 0.001; *I*^2^ = 99.70%) followed by *Toxoplasma gondii* (21.93%; 6.23–37.61%; *p* < 0.001; *I*^2^ = 99.30%), hepatitis E virus (18.29%; 11.72–24.86%; *p* < 0.001; *I*^2^ = 96.70%), *Escherichia coli* (16.34%; 13.08–19.59%; *p* < 0.001; *I*^2^ = 98.60%), *Campylobacter* spp. (8.09%; 3.48–12.70%; *p* < 0.001; *I*^2^ = 97.70%), *Salmonella* spp. (7.77%; 4.74–10.79%; *p* < 0.001; *I*^2^ = 94.10%), *Cryptosporidium* spp. (6.61%; 0.25–12.97%; *p* < 0.001; *I*^2^ = 98.60%), *Giardia duodenalis* (2.88%; 2.26–3.50%; *p* < 0.001; *I*^2^ = 95.10%), *Schistosoma* sp. (2.05%; 0.83–3.27%; *p* < 0.001; *I*^2^ = 99.00%), *Trichuris trichiura* (1.48%; 1.05–1.92%; *p* < 0.001; *I*^2^ = 97.50%), *Entamoeba histolytica/dispar* (0.62%; 0.366–0.87%; *p* < 0.001; *I*^2^ = 88.20%), *Hymenolepis nana* (0.21%; 0.12–0.31%; *p* < 0.001; *I*^2^ = 82.10%), and *Taenia* spp. (0.10%; −0.03–0.24%; *p* = 0.02; *I*^2^ = 74.30%). The overall prevalence by meta-analysis showed that bacterial organisms were the major group of pathogens followed by parasitic and viral pathogens.

### 3.4. The Pathogens at the Human—Animal–Environmental Interface

**Figure 4:**
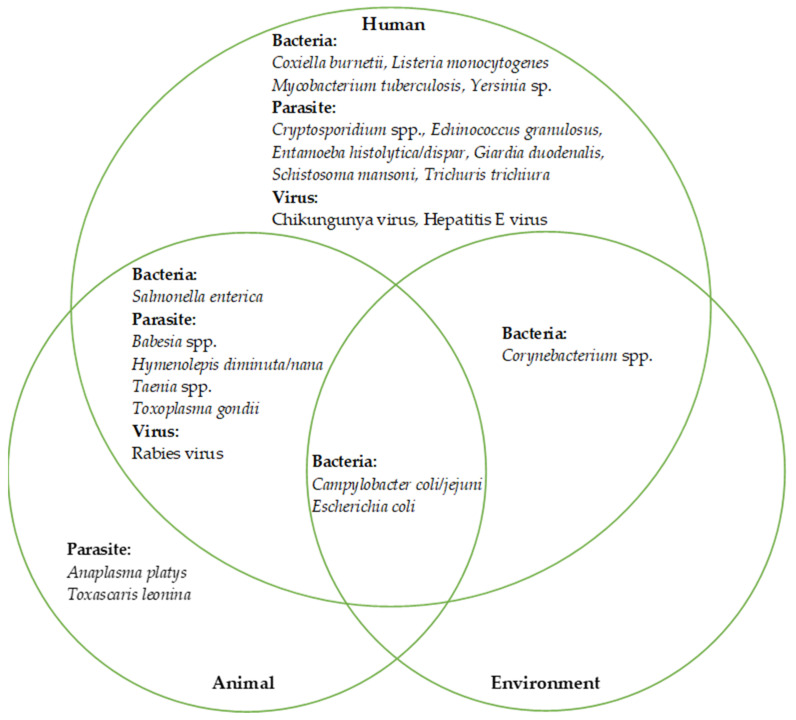
Rodent-related zoonotic pathogens identified at the human–animal–environmental interface in Qatar.

Of the 88 rodent-borne disease pathogens listed by mini-review at the beginning of the current systematic review, we identified 23 disease pathogens in Qatar. We described the interface of these pathogens in terms of humans, animals, and environmental hosts to determine the relationship with the One Health process in Qatar (Figure 4): we found 12 parasitic, 8 bacterial, and 3 viral pathogens. Our review revealed that *Campylobacter* spp. (including *Campylobacter coli* and *Campylobacter jejuni*) and *E. coli* are common in humans, animals, and the environment. *Salmonella* spp. (mainly *Salmonella enterica*), *Babesia* spp., *Taenia* spp., *T. gondii*, and rabies virus were reported from humans and animals. *Corynebacterium* spp. was the only pathogen reported from both humans and the environment.

*M. tuberculosis* was detected within various forms in humans of Qatar, such as abdominal tuberculosis (TB), mastitis pulmonary TB, pleural TB, peritoneal TB, ocular TB, pancreatic TB, spinal TB, tuberculous adenitis, tuberculous arthritis, tuberculous meningitis, tuberculous peritonitis, military TB, latent TB, and multi-drug resistant TB. Moreover, childhood TB with many of the above forms has been detected in children in Qatar.

Supplementary Table S2 shows that the pathogenic *E. coli, Salmonella* spp., and *Campylobacter* spp. are three frequently reported causes of human gastroenteritis in this country. *E. coli*, including EAEC (enteroaggregative *E. coli*), EIEC (enteroinvasive *E. coli*), EPEC (enteropathogenic *E. coli*: EPEC 2, EPEC 3, and EPEC 4), ETEC (enterotoxigenic *E. coli*), STEC (Shiga-like toxin-producing *E. coli*), and *E. coli* O157: H7 were detected among humans. Besides human gastroenteritis, *E. coli* was found to cause surgical wound infection, arthritis, genital tract infection, meningitis, peritonitis, pneumonia, septicemia, skin infection, and urinary tract infection. *E. coli* O (O157: H7, O26, O45, O103, and O111) was identified from animal sources, such as camel, cattle, and sheep. *E. coli* was isolated in human food, such as fresh fruit juice; fresh vegetables; cattle and camel milk; meat animal carcasses, such as camel, cattle, chicken, and sheep carcasses; hand swabs of fresh product market workers; market environments; animal bedding; feed and water troughs; and abattoir environments.

*S. enterica* (type B, C1, C2, D, E), *S. enterica* paratyphi A, *S. enterica* Typhi were detected in humans. Among the non-human sources, *S. enterica* was isolated from animal bedding, camel carcasses, and cattle feces. In addition, *Campylobacter* spp., such as *C. coli, C. fetus, C. jejuni, C. laridis,* and *C. upsaliensis*, were confirmed from human diarrheagenic samples. Moreover, *C. coli* and *C. jejuni* were isolated from non-human sources, via camel and cattle milk; camel, cattle, and sheep feces; camel, cattle, chicken, and sheep carcasses; cattle udders; chicken abattoirs; feed water troughs; and bedding of animals in livestock farms.

*Corynebacterium* spp. was isolated from a fresh product market. *Listeria monocytogenes* was confirmed in children (<1 year), causing meningitis. In addition, soldiers at the US army base in Qatar were positive for *Coxiella burnetii* antibodies, and non-specific *Yersinia* was detected in fecal samples of humans with gastroenteritis.

The parasites that were detected in Qatar included 5 protozoa, 3 cestodes, 2 nematodes, and one trematode. *T. gondii* was reported to have vertically transmitted from mother to baby. Diarrheagenic protozoa, such as *Cryptosporidium parvum, Cryptosporidium hominis, Cryptosporidium meleagridis, G. duodenalis*, and pathogenic amoebae (*Entamoeba histolytica/dispar*), were reported among humans in Qatar. Besides gastroenteritis, *E. histolytica* was detected to cause a liver abscess. There was a case of non-specific human babesiosis in Qatar. However, *Babesia gibsoni* and *Babesia vogeli* are present among pet dogs in this country. Among the cestodes, *Hymenolepis diminuta* is a common parasite among rodents.

*H. nana* and *Echinococcus granulosus* were reported in humans. Non-specific *Taenia* and *Taenia taeniaeformis* were identified in humans and cats, respectively. Among the nematodes, *Toxascarsis leonina* was identified in cats, and *T. trichuria* was identified in humans. However, only the trematode *Schistosoma mansoni* was reported among humans in this country. The review found three viruses in Qatar, including chikungunya, hepatitis E, and rabies, of which rabies was reported in humans, camels, and foxes.

## 4. Discussion

### 4.1. Characteristics of Rodent-Borne Pathogens

The current review studied 94 research articles to understand the rodent-related zoonotic pathogens in Qatar. About 25% (23/88) of the rodent-related pathogens have been reported in this country. Most of the pathogens (20/23) were from humans, whereas only *H. diminuta* was from rodents. However, all these 23 infectious agents are important as they are zoonotic and can cross the species barrier at any time. In addition, some infectious agents have higher importance for public health in Qatar, such as *T. gondii*, *S. enterica*, which were reported multiple times or from multiple sources.

### 4.2. Bacterial Pathogens

We detected different types of bacterial pathogens in the current review. *M. tuberculosis* was the most studied pathogen, followed by *E. coli*, *Salmonella* spp., and *Campylobacter* spp. The overall estimated pooled prevalence (30.89%) suggests that tuberculosis is a high-risk disease in this country. However, the reviewed studies tested tuberculosis mostly among the suspected cases, which may not represent the population of Qatar. Rodents act as a reservoir of *Mycobacterium microti*, a member of the *M. tuberculosis* complex [123,124,125]. *M. microti* was not detected in rodents or humans in Qatar. Therefore, the rodent role in TB prevalence in Qatar remains to be confirmed. Previous studies suggested that immigrant workers can be a source of TB in Qatar [53], as TB is more prevalent among immigrants, especially newly arrived persons [37,40,43,65]. The review showed that *E. coli*, *Salmonella* spp., and *Campylobacter* spp. are the leading causes of human gastroenteritis in Qatar [121]. Pathogenic *E. coli*, *S. enterica*, *C. coli/jejuni* were reported from non-human samples [76,82,102,121]. Rodent can mediate these food-borne pathogens to humans and animals by contaminating the foods and water [5,126,127,128]. Enteric fever by *S. enterica* serovar Typhi was considered a border disease in Qatar, imported from the endemic countries, such as Bangladesh, India, Pakistan, and Nepal by immigrant workers [31,78]. *R. norvegicus* from the wholesale market of Doha was found to carry the oriental rat flea *Xenopsylla astia* [21,22]. *Xenopsylla astia* is a carrier of *Bartonella* spp., *Coxiella burnetti,* and *Yersinia pestis* [129,130,131].

### 4.3. Parasitic Pathogens

The largest group of rodent-related pathogens in the current review was parasites, of which *T. gondii* was the most prevalent among humans in Qatar. *T. gondii* was reported with a vertical transmission from mother to fetus [50]. Besides free-living cats, *T. gondii* was detected in cheetahs [55]. Rodents might be involved with the transmission of *T. gondii* in Qatar, which needs to be confirmed. Qatar residents from Africa showed higher infection indices with *T. gondii*, *H. nana,* and *Taenia* spp. than did the residents from Asia [49,85]. *Cryptosporidium* spp., *H. nana,* and *Taenia* spp. are more prevalent in newly arrived residents [60,85,101]. Pathogenic amoebiasis are more prevalent among the immigrants from Asia than in those from Africa and other Arab countries [83,84,85]. Trichuriasis is mostly prevalent among residents from Asia [61,86], particularly from Eastern Asian countries [85], such as the Philippines and Indonesia [52]. Furthermore, cerebral schistosomiasis was reported in Filipino residents living in Qatar [122]. However, there is an information gap regarding rodent-borne diseases in humans, rodents, other animals, and the environmental interface in Qatar. *H. diminuta* was reported in *R. norvegicus* [21,22] with no report among humans. On the other hand, *H. nana* was reported from humans but not from rodents or other animals. In addition, the studies that identified rodent-related pathogens in animals and environmental sources may not represent the overall scenario at the non-human facilities in Qatar.

### 4.4. Viral Pathogens

Out of the three viruses identified in the current review, Hepatitis E showed high prevalence. Studies showed that hepatitis E in Qatar is imported by expatriates [56,90,118]. One study showed that Nepal could be a significant source of hepatitis E in Qatar [56]. Nepal is a hyperendemic country for the hepatitis E virus, where commensal rodents were found positive with hepatitis E virus [132]. Human cases of rabies in Qatar were confirmed in immigrants from Nepal [119]. Previous reports showed that rodents could be infected with rabies [133], with a low risk for transmitting the virus [134].

### 4.5. Possible Transmission of Rodent-Borne Pathogens at the Human–Animal–Environment Interface

The records of the Qatar Pest Control Company and the pest control unit of the Ministry of Municipality and Environments show that commensal rodents are more prevalent in livestock and agricultural farms than they are in residential, commercial, or industrial areas [135,136]. Most of the workers in these agriculture and livestock facilities are from South Asia [136]. The traditional livestock farms in Qatar are multi-species animal farms with poor biosecurity management [136,137]. A previous study showed that over 70% of the livestock farms are infested with rodents, such as *M. musculus, R. norvegicus,* and *R. rattus* [24]. In the residential area, rodents are more prevalent in bachelor accommodations [135]. It is plausible that immense ongoing efforts in urbanization and agricultural projects, in addition to climate change [138,139,140], may be conducive to a species-jump of rodent-borne pathogens from immigrant workers to livestock animals and rodents. Further, the introduction and establishment of new rodent species and their associated vectors in Qatar can increase such potential risks. However, the reports of *E. coli, Salmonella* spp., *M. tuberculosis, Cryptosporidium* spp., *T. gondii, Giardia* spp., and *Entamoeba* spp. among the residents from different nationalities, including native Qatari and children, means that there might be an autochthonous internal and dynamic transmission of these pathogens among the community.

The seaports and maritime shipping routes of Qatar are immensely linked to many countries to import foodstuffs, animals, crops, and animal feed and fodder. As the rodents, such as *R. norvegicus* and *R. rattus,* usually live in ships used for traveling and trades of agricultural food products [141], these rodents can move between the terminus countries and Qatar and possibly can introduce unknown pathogens to Qatar. In this respect, international seaports may play a significant role in zoonotic disease spread [141,142]. The plague outbreak in Australia in 1900 [143] and Hong Kong in 1894 [144] was linked to rodent entry through the ports. Several rodent-borne disease agents reported from the central fresh product market in Qatar may indicate that pathogens are introduced into the country when fresh market products are imported from abroad. Further studies are required to find the link between the cross-border import of rodent-related pathogens through humans, animals, or agro-products spillover in Qatar.

### 4.6. Limitations

Our study is not without limitations. Some of the limitations were identified during our work. We only conducted a mini-review (at the beginning of the systematic review) to understand rodent-related zoonotic pathogens in general. In there, we emphasized only the descriptive articles [3,5,6] and 35 additional reports to list rodent-borne zoonotic diseases [123,124,127,128,133,145,146,147,148,149,150,151,152,153,154,155,156,157,158,159,160,161,162,163,164,165,166,167,168,169,170,171,172,173,174]. Therefore, there is a chance we missed pathogens that were not described by these studied articles. There has been limited rodent-related research done at the animal–environment interface in Qatar. Finally, some related information may be out of the scope of our current systematic review and meta-analysis.

## 5. Conclusions

This review showed pathogens at the human–animal–environment interface in Qatar for which rodents can become potential mediators in transmission. A total of 23 pathogens were listed, which were mostly reported from humans. *M. tuberculosis, E. coli, T. gondii*, and hepatitis E virus were the most prevalent pathogens among humans. Besides rodents, other animals such as dogs, cats, and livestock animals can be involved in the transmission cycle. However, as there is a lack of research on rodents and other animals in this country, the transmission cycle of the stated infectious agents remains unclear. Therefore, extensive studies are required to investigate rodents and rodent-borne zoonotic pathogens among the diverse human population, livestock and pet animals, rodents, and environments in various ecosystems in Qatar. Furthermore, these studies should pursue a multidisciplinary One Health approach with contributions from zoologists, ecologists, microbiologists, entomologists, veterinarians, and public health experts to understand rodent-related zoonoses in Qatar.

## Figures and Tables

**Table 1:** Characteristics of the reviewed articles.

Characteristics	Number of Articles (%; 95%CI)	References
**Publication Year**		
1991–1995	1 (1.06; 0.027–5.79)	[31]
1996–2000	3 (3.19; 0.66–9.04)	[32,33,34]
2001–2005	12 (12.77; 6.77–21.24)	[21,35,36,37,38,39,40,41,42,43,44,45]
2006–2010	13 (13.83; 7.57–22.49)	[46,47,48,49,50,51,52,53,54,55,56,57,58]
2011–2015	23 (24.47; 16.19–34.42)	[22,59,60,61,62,63,64,65,66,67,68,69,70,71,72,73,74,75,76,77,78,79,80]
2016–2020	42 (44.68; 34.41–55.29)	[81,82,83,84,85,86,87,88,89,90,91,92,93,94,95,96,97,98,99,100,101,102,103,104,105,106,107,108,109,110,111,112,113,114,115,116,117,118,119,120,121,122]
**Host**		
Humans	80 (85.11; 76.28–91.61)	[31,32,33,34,35,36,37,38,39,40,41,42,43,44,45,49,50,51,52,53,54,56,57,58,59,60,61,62,63,64,65,66,67,68,69,70,71,72,73,74,75,77,78,79,80,81,83,84,85,86,87,88,89,90,91,92,94,95,96,97,98,99,101,103,104,107,108,109,110,111,112,113,114,115,116,117,118,120,121,122]
Animals	10 (10.64; 5.22–18.70)	[21,22,46,47,48,55,82,93,105,106]	
Environment	1 (1.06; 0.027–5.79)	[36]	
Humans + Animals	1 (1.06; 0.027–5.79)	[119]
Animals + Environment	1 (1.06; 0.027–5.79)	[76]	
Humans + Environment	1 (1.06; 0.027–5.79)	[104]
**Pathogen**		
Bacteria	62 (65.96; 55.46–75.42)	[31,32,33,34,35,36,37,38,39,40,41,42,43,44,45,53,54,57,58,62,63,64,65,66,67,69,70,71,72,73,74,75,76,77,78,79,80,81,82,87,88,92,94,95,96,97,98,102,103,105,106,107,108,109,110,111,114,115,116,117,120,121]
Helminth	10 (10.64; 5.22–18.70)	[21,22,46,47,48,60,63,68,86,122]
Protozoa	9 (9.57; 4.47–17.40)	[49,50,55,83,84,89,99,100,101]
Virus	5 (5.32; 1.75–11.98)	[45,56,90,113,119]
Helminth + Protozoa	4 (4.25; 1.17–10.54)	[51,52,61,85]
Bacteria + Protozoa	4 (4.25; 1.17–10.54)	[59,91,93,112]

CI: Confidence Interval.

## Data Availability

Data sharing is not applicable to this article as no datasets were generated.

## Supplementary Materials

The following are available online at https://www.mdpi.com/article/10.3390/ijerph18115928/s1, Table S1: Prima checklist, Table S2: List of the rodent-borne zoonotic diseases; mini-review, Table S3: Extracted data from the selected 94 studies.

## References

[1] Wilson D.E., Reeder D.M. (2005). Mammal Species of the World: A Taxonomic and Geographic Reference.

[2] Burgin C.J., Colella J.P., Kahn P.L., Upham N.S. (2018). How many species of mammals are there?. J. Mammal..

[3] Rabiee M.H., Mahmoudi A., Siahsarvie R., Kryštufek B., Mostafavi E. (2018). Rodent-borne diseases and their public health importance in Iran. PLoS Negl. Trop. Dis..

[4] Khaghani R. (2007). The Economic and Health Impact of Rodent in Urban Zone and Harbours And Their Control Methods. Ann. Mil. Health Sci. Res..

[5] Meerburg B.G., Singleton G.R., Kijlstra A. (2009). Rodent-borne diseases and their risks for public health. Crit. Rev. Microbiol..

[6] Han B.A., Schmidt J.P., Bowden S.E., Drake J.M. (2015). Rodent reservoirs of future zoonotic diseases. Proc. Natl. Acad. Sci. USA.

[7] Hamidi K. (2018). How do Rodents Play Role in Transmission of Foodborne Diseases?. Nutr. Food Sci. Int. J..

[8] Strand T.M., Lundkvist Å. (2019). Rat-borne diseases at the horizon. A systematic review on infectious agents carried by rats in Europe 1995–2016. Infect. Ecol. Epidemiol..

[9] Lalis A., Leblois R., Lecompte E., Denys C., Ter Meulen J., Wirth T. (2012). The impact of human conflict on the genetics of Mastomys natalensis and Lassa virus in West Africa. PLoS ONE.

[10] Zizi M., Heyman P., Vandenvelde C. (2002). The assessment of human health risks from rodent-borne diseases by means of ecological studies of rodent reservoirs. Mil. Med..

[11] Gubler D.J., Reiter P., Ebi K.L., Yap W., Nasci R., Patz J.A. (2001). Climate variability and change in the United States: Potential impacts on vector- and rodent-borne diseases. Environ. Health Perspect..

[12] Kilpatrick A.M., Randolph S.E. (2012). Drivers, dynamics, and control of emerging vector-borne zoonotic diseases. Lancet.

[13] Mangili A., Gendreau M.A. (2005). Transmission of infectious diseases during commercial air travel. Lancet.

[14] WTG Qatar Weateher, Climate and Geography. https://www.worldtravelguide.net/guides/middle-east/qatar/weather-climate-geography/.

[15] Snoj J. Population of Qatar by Nationality—2019 Report; Priya D’Souza Communications. http://priyadsouza.com/population-of-qatar-by-nationality-in-2017/.

[16] Planning and Statistics Authority (2020). Monthly Figures on Total Population. https://www.psa.gov.qa/en/statistics1/StatisticsSite/pages/population.aspx.

[17] Planning and Statistics Authority (2018). Labor Force Sample Survey. https://www.psa.gov.qa/en/statistics/Statistical%20Releases/Social/LaborForce/2018/statistical_analysis_labor_force_2018_En.pdf.

[18] FAO Country Profile—Qatar. http://www.fao.org/3/ca0349en/CA0349EN.pdf.

[19] Miniaoui H., Irungu P., Kaitibie S. (2018). Contemporary Issues in Qatar’s Food Security.

[20] Custom Department (2021). The Annual Custom Report–2020.

[21] Abu-Madi M.A., Behnke J.M., Mikhail M., Lewis J.W., Al-Kaabi M.L. (2005). Parasite populations in the brown rat Rattus norvegicus from Doha, Qatar between years: The effect of host age, sex and density. J. Helminthol..

[22] Abu-Madi M.A., Lewis J.W., Mikhail M., El-Nagger M.E., Behnke J.M. (2001). Monospecific helminth and arthropod infections in an urban population of brown rats from Doha, Qatar. J. Helminthol..

[23] Al Thani M.A. (2015). Irkaya.

[24] Noureldin E.M., Farrag H. (2010). Rodent control strategy in animal farms (izzab) in Qatar. Qatar Foundation Annual Research Forum Proceedings.

[25] Himsworth C.G., Parsons K.L., Jardine C., Patrick D.M. (2013). Rats, cities, people, and pathogens: A systematic review and narrative synthesis of literature regarding the ecology of rat-associated zoonoses in urban centers. Vector Borne Zoonotic Dis..

[26] World Health Organization (2019). Status of Endemicity of Cutaneous Leishmaniasis. https://apps.who.int/neglected_diseases/ntddata/leishmaniasis/leishmaniasis.html.

[27] Public Health England (2020). Guidance: Rabies Risks in Terrestrial Animals by Country. https://www.gov.uk/government/collections/rabies-risk-assessment-post-exposure-treatment-management.

[28] Centers for Disease Control and Prevention (2020). Travelers’ Health.

[29] Moher D., Liberati A., Tetzlaff J., Altman D.G. (2009). Preferred reporting items for systematic reviews and meta-analyses: The PRISMA statement. PLoS Med..

[30] Higgins J.P., Thompson S.G. (2002). Quantifying heterogeneity in a meta—Analysis. Stat. Med..

[31] Uwaydah A.K., Matar I., Chacko K.C., Davidson J.C. (1991). The emergence of antimicrobial resistant Salmonella typhi in Qatar: Epidemiology and therapeutic implications. Trans. Royal Soc. Trop. Med. Hyg..

[32] Al-Marri M.R., Almosleh A., Almoslmani Y. (2000). Primary tuberculosis of the breast in Qatar: Ten year experience and review of the literature. Eur. J. Surg..

[33] Al-Marri M., Kirkpatrick M.B. (2000). Erythrocyte sedimentation rate in childhood tuberculosis: Is it still worthwhile?. Int. J. Tuberc. Lung Dis..

[34] Szmiegielski W., Venkataraman B., Ejeckam G.C., Jarikre L.N. (1998). Abdominal tuberculosis in Qatar: A clinico-radiological study. Int. J. Tuberc. Lung Dis..

[35] Al Marri M.R.H.A., Al Qatami M., Al Janahi M. (2002). The tuberculin skin test in children with tuberculosis in the State of Qatar. Qatar Med J..

[36] Al-Jedah J.H., Robinson R.K. (2002). Nutritional value and microbiological safety of fresh fruit juices sold through retail outlets in Qatar. Pak. J. Nutr..

[37] Al-Khal A.L., Bener A., Enarson D.A. (2005). Tuberculosis among garment workers in an Arabian developing country: State of Qatar. Arch. Environ. Occup. Health.

[38] Al-Marri M. (2001). Childhood tuberculosis in the State of Qatar: The effect of a limited expatriate screening programme on the incidence of tuberculosis. Int. J. Tuberc. Lung Dis..

[39] Al-Marri M. (2001). Pattern of mycobacterial resistance to four anti-tuberculosis drugs in pulmonary tuberculosis patients in the State of Qatar after the implementation of DOTS and a limited expatriate screening programme. Int. J. Tuberc. Lung Dis..

[40] Alsoub H., Al Alousi F.S. (2001). Miliary tuberculosis in Qatar: A review of 32 adult cases. Ann. Saudi Med..

[41] Howady F.S., Al Soub H., Al Khal A.L. (2003). Spinal tuberculosis in Qatar. Qatar Med. J..

[42] Ibrahim A.S., Allangawi M.H., Sattar H.A., Mobyed H.S., Almohammed A.A. (2005). Indications, diagnostic yields and complications of transbronchial biopsy over 5 years in the State of Qatar. Saudi Med. J..

[43] Ibrahim W.H., Ghadban W., Khinji A., Yasin R., Soub H., Al-Khal A.L., Bener A. (2005). Does pleural tuberculosis disease pattern differ among developed and developing countries. Respir. Med..

[44] Mobayedh H.M.S., Sattar H.A., Ghadban W.K., Al Khal A.L., Al Soub H., Al Alousi F.S., Al Mohammed A.A. (2002). Diagnostic value of fiberoptic bronchoscopy in suspected pulmonary tuberculosis in the state of Qatar. Qatar Med. J..

[45] Nahman A., Hammoudeh M. (2003). Pyogenic arthritis in Qatar. Qatar Med. J..

[46] Abu-Madi M.A., Al-Ahbabi D.A., Al-Mashhadani M.M., Al-Ibrahim R., Pal P., Lewis J.W. (2007). Patterns of parasitic infections in faecal samples from stray cat populations in Qatar. J. Helminthol..

[47] Abu-Madi M.A., Pal P., Al-Thani A., Lewis J.W. (2008). Descriptive epidemiology of intestinal helminth parasites from stray cat populations in Qatar. J. Helminthol..

[48] Abu-Madi M.A., Behnke J.M., Prabhaker K.S., Al-Ibrahim R., Lewis J.W. (2010). Intestinal helminths of feral cat populations from urban and suburban districts of Qatar. Vet. Parasitol..

[49] Abu-Madi M.A., Al-Molawi N., Behnke J.M. (2008). Seroprevalence and epidemiological correlates of Toxoplasma gondii infections among patients referred for hospital-based serological testing in Doha, Qatar. Parasit Vectors.

[50] Abu-Madi M.A., Behnke J.M., Dabritz H.A. (2010). Toxoplasma gondii seropositivity and co-infection with TORCH pathogens in high-risk patients from Qatar. Am. J. Trop. Med. Hyg..

[51] Abu-Madi M.A., Behnke J.M., Doiphode S.H. (2010). Changing trends in intestinal parasitic infections among long-term-residents and settled immigrants in Qatar. Parasites Vectors.

[52] Abu-Madi M.A., Behnke J.M., Ismail A. (2008). Patterns of infection with intestinal parasites in Qatar among food handlers and housemaids from different geographical regions of origin. Acta Trop..

[53] Al Marri M.R.H.A., Al Hail L., Al Otaibi S., Al Marri N.D. (2006). The time of reactivation of tuberculosis in expatriates in the State of Qatar. Qatar Med. J..

[54] Al-Aani F.K., Abusalah S., Al-Aqeedi R., Ibrahim A. (2009). Salmonella meningitis in an adult with type B viral hepatitis and an incidental schwannoma. BMJ Case Rep..

[55] Dubey J.P., Pas A., Rajendran C., Kwok O.C.H., Ferreira L.R., Martins J., Hebel C., Hammer S., Su C. (2010). Toxoplasmosis in Sand cats (Felis margarita) and other animals in the Breeding Centre for Endangered Arabian Wildlife in the United Arab Emirates and Al Wabra Wildlife Preservation, the State of Qatar. Vet. Parasitol..

[56] Ibrahim A.S., Alkhal A., Jacob J., Ghadban W., Almarri A. (2009). Hepatitis E in Qatar imported by expatriate workers from Nepal: Epidemiological characteristics and clinical manifestations. J. Med. Virol..

[57] Khan F.Y., Elshafie S.S., Almaslamani M., Abu-Khattab M., El Hiday A.H., Errayes M., Almaslamani E. (2010). Epidemiology of bacteraemia in Hamad general hospital, Qatar: A one year hospital-based study. Travel. Med. Infect. Dis..

[58] Shigidi M.M., Fituri O.M., Chandy S.K., Asim M., Al Malki H.A., Rashed A.H. (2010). Microbial spectrum and outcome of peritoneal dialysis related peritonitis in Qatar. Saudi J. Kidney Dis. Transpl..

[59] Abbas M.T., Khan F.Y., Muhsin S.A., Al-Dehwe B., Abukamar M., Elzouki A.N. (2014). Epidemiology, clinical features and outcome of liver abscess: A single reference center experience in Qatar. Oman Med. J..

[60] Abu-Madi M.A., Behnke J.M., Ismail A., Al-Olaqi N., Al-Zaher K., El-Ibrahim R. (2011). Comparison of intestinal parasitic infection in newly arrived and resident workers in Qatar. Parasit Vectors.

[61] Abu-Madi M.A., Behnke J.M., Doiphode S.H. (2013). Intestinal parasitic infections among long-term-residents and settled immigrants in Qatar in the period 2005 to 2011. Am. J. Trop. Med. Hyg..

[62] Al Alousi F.S., Abu Khattab M., Al Soub H., Al-Khal A.L., Al-Suwaidi Z.D. (2012). Value of examining 3 sputum samples in the diagnosis of active pulmonary tuberculosis in qatar. Infect. Dis. Clin. Pract..

[63] Al Ani A.M., Khan F.Y., Elzouki A.N., Al Hajri M., Ibrahim W. (2014). Epidemiology of hydatid disease in Qatar: A hospital based study from 2000 to 2013. Asian Pac. J. Trop. Med..

[64] Al Jama F.E. (2012). Risk factors for wound infection after lower segment cesarean section. Qatar Med. J..

[65] Al Marri M.R.H.A. (2012). The tuberculin skin test in confirmed pulmonary tuberculosis in the state of Qatar: Where we stand?. Qatar Med. J..

[66] Al-Mulla N.A., Taj-Aldeen S.J., El Shafie S., Janahi M., Al-Nasser A.A., Chandra P. (2014). Bacterial bloodstream infections and antimicrobial susceptibility pattern in pediatric hematology/oncology patients after anticancer chemotherapy. Infect. Drug Resist..

[67] Al-Thani A., Baris M., Al-Lawati N., Al-Dhahry S. (2013). Characterising the aetiology of severe acute gastroenteritis among patients visiting a hospital in Qatar using real-time polymerase chain reaction. BMC Infect. Dis..

[68] Derbala M., Elbadri M.E., Amer A.M., AlKaabi S., Sultan K.H., Kamel Y.M., Elsayed E.H., Avades T.Y., Chandra P., Shebl F.M. (2015). Aspartate transaminase to platelet ratio index in hepatitis C virus and Schistosomiasis coinfection. World J. Gastroenterol..

[69] Ehlayel M.S., Bener A., Laban M.A. (2013). Primary immunodeficiency diseases in children: 15 year experience in a tertiary care medical center in Qatar. J. Clin. Immunol..

[70] Garcell H.G., Ramirez E.C., Contreras A.K., Garcia F.G. (2014). Latent tuberculosis infection in healthcare workers at a community hospital in Qatar. J. Infect. Public Health.

[71] Ghunaim H., Behnke J.M., Aigha I., Sharma A., Doiphode S.H., Deshmukh A., Abu-Madi M.M. (2015). Analysis of resistance to antimicrobials and presence of virulence/stress response genes in Campylobacter isolates from patients with severe diarrhoea. PLoS ONE.

[72] Imam Y.Z.B., Ahmedullah H.S., Akhtar N., Chacko K.C., Kamran S., Al Alousi F., Alsuwaidi Z., Almaslmani M., Al Khal A.L., Deleu D. (2015). Adult Tuberculous Meningitis in Qatar: A Descriptive Retrospective Study from its Referral Center. Eur. Neurol..

[73] Khan F.Y., Al-Muzrakchi A.M., Elbedawi M.M., Al-Muzrakchi A.A., Al Tabeb A. (2012). Peritoneal tuberculosis in Qatar: A five-year hospital-based study from 2005 to 2009. Travel Med. Infect. Dis..

[74] Khan F.Y., Abu-Khattab M., Baagar K., Mohamed S.F., Elgendy I., Anand D., Malallah H., Sanjay D. (2013). Characteristics of patients with definite septic arthritis at Hamad General Hospital, Qatar: A hospital-based study from 2006 to 2011. Clin. Rheumatol..

[75] Khan F.Y., Hamza M., Omran A.H., Saleh M., Lingawi M., Alnaqdy A., Rahman M.O.A., Ahmedullah H.S., Hamza A., Al Ani A. (2013). Diagnostic value of pleural fluid interferon-gamma and adenosine deaminase in patients with pleural tuberculosis in Qatar. Int. J. Gen. Med..

[76] Mohammed H.O., Stipetic K., Salem A., McDonough P., Chang Y.F., Sultan A. (2015). Risk of Escherichia coli O157:H7, Non-O157 Shiga Toxin-Producing Escherichia coli, and Campylobacter spp. in Food Animals and Their Products in Qatar. J. Food Prot..

[77] Royal J., Riddle M.S., Mohareb E., Monteville M.R., Porter C.K., Faix D.J. (2013). Seroepidemiologic Survey for Coxiella burnetii Among US Military Personnel Deployed to Southwest and Central Asia in 2005. Am. J. Trop. Med. Hyg..

[78] Thandassery R.B., Sharma M., Abdelmola A., Derbala M.F.M., Al Kaabi S.R. (2014). Uncommon gastrointestinal complications of enteric fever in a non-endemic country. Qatar Med. J..

[79] Thomas M., AlGherbawe M. (2014). Acute myeloid leukemia presenting with pulmonary tuberculosis. Case Rep. Infect. Dis..

[80] Zowawi H.M., Sartor A.L., Balkhy H.H., Walsh T.R., Al Johani S.M., AlJindan R.Y., Alfaresi M., Ibrahim E., Al-Jardani A., Al-Abri S. (2014). Molecular characterization of carbapenemase-producing Escherichia coli and Klebsiella pneumoniae in the countries of the Gulf cooperation council: Dominance of OXA-48 and NDM producers. Antimicrob. Agents Chemother..

[81] Abdelmaguid N., Seleem W.S., Soliman A.T., Mohamed R.S., Elgharbawy F.M., Yassin H., De Sanctis V. (2019). Clinical presentations, laboratory analysis and linear growth in 50 neonates and young infants with acute meningitis: One year experience of a single center in Qatar. Mediterr. J. Hematol. Infect. Dis..

[82] Abu-Madi M., Behnke J.M., Sharma A., Bearden R., Al-Banna N. (2016). Prevalence of Virulence/Stress Genes in Campylobacter jejuni from Chicken Meat Sold in Qatari Retail Outlets. PLoS ONE.

[83] Abu-Madi M., Boughattas S., Behnke J.M., Sharma A., Ismail A. (2017). Coproscopy and molecular screening for detection of intestinal protozoa. Parasites Vectors.

[84] Abu-Madi M.A., Behnke J.M., Boughattas S., Al-Thani A., Doiphode S.H. (2016). A decade of intestinal protozoan epidemiology among settled immigrants in Qatar. BMC Infect. Dis..

[85] Abu-Madi M.A., Behnke J.M., Ismail A., Boughattas S. (2016). Assessing the burden of intestinal parasites affecting newly arrived immigrants in Qatar. Parasit Vectors.

[86] Abu-Madi M.A., Behnke J.M., Boughattas S., Al-Thani A., Doiphode S.H., Deshmukh A. (2016). Helminth infections among long-term-residents and settled immigrants in Qatar in the decade from 2005 to 2014: Temporal trends and varying prevalence among subjects from different regional origins. Parasites Vectors.

[87] Ahmed M.A.S., Bansal D., Acharya A., Elmi A.A., Hamid J.M., Ahmed A.M.S., Chandra P., Ibrahim E., Sultan A.A., Doiphode S. (2016). Antimicrobial susceptibility and molecular epidemiology of extended-spectrum beta-lactamase-producing Enterobacteriaceae from intensive care units at Hamad Medical Corporation, Qatar. Antimicrob. Resist. Infect. Control..

[88] Ahmedullah H., Khan F.Y., Al Maslamani M., Al Soub H., Chacko K., Abu Khattab M., Mahmoud S., Howaidy F., Thapur M., Al Madhoun E. (2018). Epidemiological and Clinical Features of Salmonella Typhi Infection Among Adult Patients in Qatar: A Hospital-based Study. Oman Med. J..

[89] Al Soub H., Al Maslamani M., Ahmedullah H.S., Shawkat A., Ibrahim F.A., Kanbar N.A. (2016). First case of babesiosis in Qatar: Case report. Jordan Med. J..

[90] Al-Absi E.S., Al-Sadeq D.W., Younis M.H., Yassine H.M., Abdalla O.M., Mesleh A.G., Hadwan T.A., Amimo J.O., Thalib L., Nasrallah G.K. (2018). Performance evaluation of five commercial assays in assessing seroprevalence of HEV antibodies among blood donors. J. Med. Microbiol..

[91] Al-Baker Z.M., Bodaghi B., Khan S.A. (2018). Clinical Patterns and Causes of Uveitis in a Referral Eye Clinic in Qatar. Ocular Immunol. Inflamm..

[92] Al-Dahshan A., Elyamani R., Naja S., Chehab M., Nour M., Elmagboul E., Saleh T., Al-Romaihi H., Farag E. (2019). Epidemiological characteristics of a salmonella outbreak among infants in Qatar, 2017. Qatar Med. J..

[93] Alho A.M., Lima C., Latrofa M.S., Colella V., Ravagnan S., Capelli G., de Carvalho L.M., Cardoso L., Otranto D. (2017). Molecular detection of vector-borne pathogens in dogs and cats from Qatar. Parasites Vectors.

[94] Ali M., Shaukat A., Ai-Suwaidi Z., Al-Maslamani M. (2019). Tuberculosis of Pancreas, the First Case Reported from Qatar. Int. J. Mycobacteriol..

[95] Al-Nesf M.A., Jerobin J., Al-Alawi A.A., El-Kassim M., Mobayed H., Mohammed T.R.N. (2019). Etiology and outcome of hemoptysis in Qatar, a high-resource country with a large expatriate population: A retrospective study. Qatar Med. J..

[96] Al-Shaer M.H., Mansour H., Elewa H., Salameh P., Iqbal F. (2017). Treatment outcomes of fixed-dose combination versus separate tablet regimens in pulmonary tuberculosis patients with or without diabetes in Qatar. BMC Infect. Dis..

[97] Al-Shaer M.H., Elewa H., Alkabab Y., Nazer L.H., Heysell S.K. (2018). Fixed-dose combination associated with faster time to smear conversion compared to separate tablets of anti-tuberculosis drugs in patients with poorly controlled diabetes and pulmonary tuberculosis in Qatar. BMC Infect. Dis..

[98] Ben Abid F., Abdel Rahman S.A.S.H. (2018). A case report of TB versus idiopathic granulomatous mastitis with erythema nodosum, reactive arthritis, cough, and headache. Aging Male.

[99] Boughattas S., Behnke J.M., Al-Ansari K., Sharma A., Abu-Alainin W., Al-Thani A., Abu-Madi M.A. (2017). Molecular Analysis of the Enteric Protozoa Associated with Acute Diarrhea in Hospitalized Children. Front. Cell. Infect. Microbiol..

[100] Boughattas S., Behnke J., Sharma A., Abu-Madi M. (2017). Seroprevalence of Toxoplasma gondii infection in feral cats in Qatar. BMC Vet. Res..

[101] Boughattas S., Behnke J.M., Al-Sadeq D., Ismail A., Abu-Madi M. (2019). Cryptosporidium spp., prevalence, molecular characterisation and socio-demographic risk factors among immigrants in Qatar. PLoS Negl. Trop. Dis..

[102] Chang Y.C., Scaria J., Ibraham M., Doiphode S., Chang Y.-F., Sultan A., Mohammed H.O. (2016). Distribution and factors associated with Salmonella enterica genotypes in a diverse population of humans and animals in Qatar using multi-locus sequence typing (MLST). J. Infect. Public Health.

[103] Dousa K.M., Hamad A., Albirair M., Al Soub H., Elzouki A.N., Alwakeel M.I., Thiel B.A., Johnson J.L. (2019). Impact of Diabetes Mellitus on the Presentation and Response to Treatment of Adults With Pulmonary Tuberculosis in Qatar. Open Forum Infect. Dis..

[104] El-Nemr I.M., Mushtaha M., Sundararaju S., Fontejon C., Suleiman M., Tang P., Goktepe I., Hasan M.R. (2019). Application of MALDI Biotyper System for Rapid Identification of Bacteria Isolated from a Fresh Produce Market. Curr. Microbiol..

[105] Eltai N., Al Thani A.A., Al-Hadidi S.H., Abdfarag E.A., Al-Romaihi H., Mahmoud M.H., Alawad O.K., Yassine H.M. (2020). Antibiotic resistance profile of commensal Escherichia coli isolated from healthy sheep in Qatar. J. Infect. Dev. Ctries.

[106] Eltai N.O., Abdfarag E.A., Al-Romaihi H., Wehedy E., Mahmoud M.H., Alawad O.K., Al-Hajri M.M., Al Thani A.A., Yassine H.M. (2018). Antibiotic Resistance Profile of Commensal Escherichia coli Isolated from Broiler Chickens in Qatar. J. Food Prot..

[107] Eltai N.O., Al Thani A.A., Al-Ansari K., Deshmukh A.S., Wehedy E., Al-Hadidi S.H., Yassine H.M. (2018). Molecular characterization of extended spectrum beta -lactamases enterobacteriaceae causing lower urinary tract infection among pediatric population. Antimicrob. Resist. Infect. Control..

[108] Eltai N.O., Yassine H.M., Al Thani A.A., Abu Madi M.A., Ismail A., Ibrahim E., Alali W.Q. (2018). Prevalence of antibiotic resistant Escherichia coli isolates from fecal samples of food handlers in Qatar. Antimicrob. Resist. Infect. Control..

[109] Eltai N.O., Al Thani A.A., Al Hadidi S.H., Al Ansari K., Yassine H.M. (2020). Antibiotic resistance and virulence patterns of pathogenic Escherichia coli strains associated with acute gastroenteritis among children in Qatar. BMC Microbiol..

[110] Farag E., Garcell H.G., Ganesan N., Ahmed S.N., Al-Hajri M., Al Thani S.M., Al-Marri S.A., Ibrahim E., Al-Romaihi H.E. (2016). A retrospective epidemiological study on the incidence of salmonellosis in the State of Qatar during 2004–2012. Qatar Med. J..

[111] Garcell H.G., Arias A.V., Sandoval C.A., Garcia E.G., Gamboa M.E., Sado A.B., Serrano R.N. (2017). Incidence and Etiology of Surgical Site Infections in Appendectomies: A 3-Year Prospective Study. Oman Med. J..

[112] Humphrey J.M., Ranbhise S., Ibrahim E., Al-Romaihi H.E., Farag E., Abu-Raddad L.J., Glesby M.J. (2016). Multiplex Polymerase Chain Reaction for Detection of Gastrointestinal Pathogens in Migrant Workers in Qatar. Am. J. Trop. Med. Hyg..

[113] Humphrey J.M., Al-Absi E.S., Hamdan M.M., Okasha S.S., Al-Trmanini D.M., El-Dous H.G., Dargham S.R., Schieffelin J., Abu-Raddad L.J., Nasrallah G.K. (2019). Dengue and chikungunya seroprevalence among Qatari nationals and immigrants residing in Qatar. PLoS ONE.

[114] Ibrahim W.H., Alousi F.H., Al-Khal A., Bener A., AlSalman A., Aamer A., Khaled A., Raza T. (2016). Diagnostic Delay among Adults with Pulmonary Tuberculosis in a High Gross Domestic Product Per Capita Country: Reasons and Magnitude of the Problem. Int. J. Prev. Med..

[115] Khan F.Y., Abu-Khattab M., Almaslamani E.A., Hassan A.A., Mohamed S.F., Elbuzdi A.A., Elmaki N.Y., Anand D., Sanjay D. (2017). Acute Bacterial Meningitis in Qatar: A Hospital-Based Study from 2009 to 2013. BioMed Res. Int..

[116] Khan F.Y. (2009). Clinical pattern of tuberculous adenitis in Qatar: Experience with 35 patients. Scand. J. Infect. Dis..

[117] Minisha F., Mohamed M., Abdulmunem D., El Awad S., Zidan M., Abreo M., Ahmad S., Fender G. (2019). Bacteriuria in pregnancy varies with the ambiance: A retrospective observational study at a tertiary hospital in Doha, Qatar. J. Perinat Med..

[118] Nasrallah G.K., Al Absi E.S., Ghandour R., Ali N.H., Taleb S., Hedaya L., Ali F., Huwaidy M., Husseini A. (2017). Seroprevalence of hepatitis E virus among blood donors in Qatar (2013–2016). Transfusion.

[119] Oude Munnink B.B., Farag E.A.B.A., GeurtsvanKessel C., Schapendonk C., van der Linden A., Kohl R., Arron G., Ziglam H., Goravey W.G.M., Coyle P.V. (2020). First molecular analysis of rabies virus in Qatar and clinical cases imported into Qatar, a case report. Int. J. Infect. Dis..

[120] Thomas M., Ibrahim W.H., Raza T., Mushtaq K., Arshad A., Ahmed M., Taha S., Al Sarafandi S., Karim H., Abdul-sattar H.A. (2017). Medical thoracoscopy for exudative pleural effusion: An eight-year experience from a country with a young population. BMC Pulm. Med..

[121] Weam B., Abraham M., Doiphode S., Peters K., Ibrahim E., Sultan A., Mohammed H.O. (2016). Foodborne Bacterial Pathogens Associated with the Risk of Gastroenteritis in the State of Qatar. Int. J. Health Sci..

[122] Zaqout A., Abid F.B., Murshed K., Al-Bozom I., Al-Rumaihi G., Al Soub H., Al Maslamani M., Al Khal A. (2019). Cerebral schistosomiasis: Case series from Qatar. Int. J. Infect. Dis..

[123] Behr M.A., Gagneux S., Tibayrenc M. (2011). 24—The Rise and Fall of the Mycobacterium tuberculosis Complex. Genetics and Evolution of Infectious Disease.

[124] Moradi E., Mosavari N., Tebianian M., Tadayon K., Ghaderi R., Soleymani Babadi K., Mohammad Taheri M., Dashtipour S., Loni R., Moradi Garavand M. (2015). Pest rodents as the essential elements of Mycobacterium bovis controlling programs. Int. J. Mycobacteriol..

[125] Cavanagh R., Begon M., Bennett M., Ergon T., Graham I.M., De Haas P.E.W., Hart C.A., Koedam M., Kremer K., Lambin X. (2002). Mycobacterium microti infection (vole tuberculosis) in wild rodent populations. J. Clin. Microbiol..

[126] Blanco Crivelli X., Rumi M.V., Carfagnini J.C., Degregorio O., Bentancor A.B. (2012). Synanthropic rodents as possible reservoirs of shigatoxigenic Escherichia coli strains. Front. Cell Infect. Microbiol..

[127] Meerburg B.G., Jacobs-Reitsma W.F., Wagenaar J.A., Kijlstra A. (2006). Presence of Salmonella and Campylobacter spp. in wild small mammals on organic farms. Appl. Environ. Microbiol..

[128] Meerburg B.G., Kijlstra A. (2007). Role of rodents in transmission of Salmonella and Campylobacter. J. Sci. Food Agric..

[129] Loftis A.D., Reeves W.K., Szumlas D.E., Abbassy M.M., Helmy I.M., Moriarity J.R., Dasch G.A. (2006). Surveillance of Egyptian fleas for agents of public health significance: Anaplasma, bartonella, coxiella, ehrlichia, rickettsia, and Yersinia pestis. Am. J. Trop. Med. Hyg..

[130] Morick D., Baneth G., Avidor B., Kosoy M.Y., Mumcuoglu K.Y., Mintz D., Eyal O., Goethe R., Mietze A., Shpigel N. (2009). Detection of Bartonella spp. in wild rodents in Israel using HRM real-time PCR. Vet. Microbiol..

[131] Nekouie H., Razavi M., Seyedipoor G. (2003). Investigation of Yersinia pestis in Xenopsylla astia. Southeast Asian J. Trop. Med. Public Health.

[132] He J., Innis B.L., Shrestha M.P., Clayson E.T., Scott R.M., Linthicum K.J., Musser G.G., Gigliotti S.C., Binn L.N., Kuschner R.A. (2002). Evidence that rodents are a reservoir of hepatitis E virus for humans in Nepal. J. Clin. Microbiol..

[133] Fitzpatrick J.L., Dyer J.L., Blanton J.D., Kuzmin I.V., Rupprecht C.E. (2014). Rabies in rodents and lagomorphs in the United States, 1995–2010. J. Am. Vet. Med. Assoc..

[134] Rohde R., Wilson P. (2015). 8 Things You May Not Know About Rabies—But Should!.

[135] Qatar Pest Control Company (2021). Rodent trapping catelogue. Semco Building, Behind HSBC Building, Airport Road.

[136] Social & Economic Survey Research Institute (2021). Priminiary Records of Agricultural Census.

[137] Farag E., Sikkema R.S., Vinks T., Islam M.M., Nour M., Al-Romaihi H., Al Thani M., Atta M., Alhajri F.H., Al-Marri S. (2018). Drivers of MERS-CoV Emergence in Qatar. Viruses.

[138] Ben Hassen T., El Bilali H., Al-Maadeed M. (2020). Agri-Food Markets in Qatar: Drivers, Trends, and Policy Responses. Sustainability.

[139] Boussaa D. (2014). Al Asmakh historic district in Doha, Qatar: From an urban slum to living heritage. J. Archit. Conserv..

[140] Karanisa T., Amato A., Richer R., Abdul Majid S., Skelhorn C., Sayadi S. (2021). Agricultural Production in Qatar’s Hot Arid Climate. Sustainability.

[141] Kuo C.-C., Wardrop N., Chang C.-T., Wang H.-C., Atkinson P.M. (2017). Significance of major international seaports in the distribution of murine typhus in Taiwan. PLOS Negl. Trop. Dis..

[142] White C.F. (1935). Plague: Modern Preventive Measures in Ships and Ports: (Section of Tropical Diseases and Parasitology). Proc. R Soc. Med..

[143] Banks P., Cleary G., Dickman C. (2011). Sydney’s bubonic plague outbreak 1900-1910: A disaster for foreshore wildlife?. Aust. Zool..

[144] Sonne O. (2016). Plague, rats, and ships The realisation of the infection routes of plague. Dan Medicinhist Arbog.

[145] Buller R.M.L., Cohen J., Powderly W.G., Opal S.M. (2017). 170—Poxviruses. Infectious Diseases.

[146] Cao C., Liang L., Wang W., Luo H., Huang S., Liu D., Xu J., Henk D.A., Fisher M.C. (2011). Common reservoirs for Penicillium marneffei infection in humans and rodents, China. Emerg. Infect. Dis..

[147] Da Costa Cordeiro H., de Vasconcelos Melo F.T., Giese E.G., Santos J.N.D. (2018). Gongylonema Parasites of Rodents: A Key to Species and New Data on Gongylonema neoplasticum. J. Parasitol..

[148] Da Silva A.M. (2010). Human echinococcosis: A neglected disease. Gastroenterol. Res. Pr..

[149] Deplazes P., Eichenberger R.M., Grimm F. (2019). Wildlife-transmitted Taenia and Versteria cysticercosis and coenurosis in humans and other primates. Int. J. Parasitol. Parasites Wildl..

[150] Dworkin M.S., Schwan T.G., Anderson D.E., Borchardt S.M. (2008). Tick-borne relapsing fever. Infect. Dis. Clin..

[151] Edmonds P., Patton C.M., Griffin P.M., Barrett T.J., Schmid G.P., Baker C.N., Lambert M.A., Brenner D.J. (1987). Campylobacter hyointestinalis associated with human gastrointestinal disease in the United States. J. Clin. Microbiol..

[152] Emmons C.W., Ashburn L.L. (1948). Histoplasmosis in Wild Rats: Occurrence and Histopathology. Public Health Rep..

[153] Favacho A.R.d.M., Andrade M.N., de Oliveira R.C., Bonvicino C.R., D’Andrea P.S., de Lemos E.R.S. (2015). Zoonotic Bartonella species in wild rodents in the state of Mato Grosso do Sul, Brazil. Microbes Infect..

[154] García-Livia K., Martín-Alonso A., Foronda P. (2020). Diversity of Cryptosporidium spp. in wild rodents from the Canary Islands, Spain. Parasit Vectors.

[155] Gillespie S.H., Pearson R.D. (2010). Principles and Practice of Clinical Parasitology.

[156] Gonçalves L.R., Favacho A.R.d.M., Roque A.L.R., Mendes N.S., Fidelis Junior O.L., Benevenute J.L., Herrera H.M., Andrea P.S., de Lemos E.R.S., Machado R.Z. (2016). Association of Bartonella Species with Wild and Synanthropic Rodents in Different Brazilian Biomes. Appl. Environ. Microbiol..

[157] Gravinatti M.L., Barbosa C.M., Soares R.M., Gregori F. (2020). Synanthropic rodents as virus reservoirs and transmitters. Rev. Soc. Bras. Med. Trop..

[158] Hildebrand J., Adamczyk M., Laskowski Z., Zaleśny G. (2015). Host-dependent morphology of Isthmiophora melis (Schrank, 1788) Luhe, 1909 (Digenea, Echinostomatinae)--morphological variation vs. molecular stability. Parasit Vectors.

[159] Kawahara M., Ito T., Suto C., Shibata S., Rikihisa Y., Hata K., Hirai K. (1999). Comparison of Ehrlichia muris Strains Isolated from Wild Mice and Ticks and Serologic Survey of Humans and Animals with E. muris as Antigen. J. Clin. Microbiol..

[160] Lv C., Zhang L., Wang R., Jian F., Zhang S., Ning C., Wang H., Feng C., Wang X., Ren X. (2009). Prevalence and molecular characterization of Cryptosporidium spp. in wild, laboratory, and pet rodents in China. Appl. Environ. Microbiol..

[161] Macnish M.G., Ryan U.M., Behnke J.M., Thompson R.C. (2003). Detection of the rodent tapeworm Rodentolepis (=Hymenolepis) microstoma in humans. A new zoonosis?. Int. J. Parasitol..

[162] Möhl K., Grosse K., Hamedy A., Wüste T., Kabelitz P., Lücker E. (2009). Biology of Alaria spp. and human exposition risk to Alaria mesocercariae-a review. Parasitol. Res..

[163] Nkogwe C., Raletobana J., Stewart-Johnson A., Suepaul S., Adesiyun A. (2011). Frequency of Detection of Escherichia coli, Salmonella spp., and Campylobacter spp. in the Faeces of Wild Rats (Rattus spp.) in Trinidad and Tobago. Vet. Med. Int..

[164] Pozio E. (2007). World distribution of Trichinella spp. infections in animals and humans. Vet. Parasitol..

[165] Scharmann W., Heller A. (2001). Survival and transmissibility of Pasteurella pneumotropica. Lab. Anim..

[166] Spickler A.R. (2020). Taeniasis, Cysticercosis, and Coenurosis. http://www.cfsph.iastate.edu/Factsheets/pdfs/taenia.pdf.

[167] Tappe D., Stich A., Frosch M. (2008). Emergence of polycystic neotropical echinococcosis. Emerg. Infect. Dis..

[168] Thomas R.J., Dumler J.S., Carlyon J.A. (2009). Current management of human granulocytic anaplasmosis, human monocytic ehrlichiosis and Ehrlichia ewingii ehrlichiosis. Expert Rev. Anti Infect. Ther..

[169] Toledo R., Esteban J.G. (2016). An update on human echinostomiasis. Trans. R Soc. Trop. Med. Hyg..

[170] Tominello T.R., Oliveira E.R.A., Hussain S.S., Elfert A., Wells J., Golden B., Ismail N. (2019). Emerging Roles of Autophagy and Inflammasome in Ehrlichiosis. Front. Immunol..

[171] Van Soolingen D., van der Zanden A.G., de Haas P.E., Noordhoek G.T., Kiers A., Foudraine N.A., Portaels F., Kolk A.H., Kremer K., van Embden J.D. (1998). Diagnosis of Mycobacterium microti infections among humans by using novel genetic markers. J. Clin. Microbiol..

[172] Vourc’h G., Halos L., Desvars A., Boué F., Pascal M., Lecollinet S., Zientara S., Duval T., Nzonza A., Brémont M. (2014). Chikungunya antibodies detected in non-human primates and rats in three Indian Ocean islands after the 2006 ChikV outbreak. Vet. Res..

[173] Wang Y., Lu L., Lan R., Salazar J.K., Liu J., Xu J., Ye C. (2017). Isolation and characterization of Listeria species from rodents in natural environments in China. Emerg. Microbes Infect..

[174] Wasiluk A. (2013). Alaria alata infection—Threating yet rarely detected trematodiasis. J. Lab. Diagn..

