# Supplementary material for: Rodent-Related Zoonotic Pathogens at the Human–Animal–Environment Interface in Qatar: A Systematic Review and Meta-Analysis
Source: Int J Environ Res Public Health. 2021 May 31;18(11):5928. doi: 10.3390/ijerph18115928 (PMC8198466; doi:10.3390/ijerph18115928)
Supplement: Supplementary file 1 [file ijerph-18-05928-s001.zip › Supplementary Table S2.pdf]

Rodent-related zoonotic pathogens at the human-animal-environmental interface in Qatar: a systematic review and meta-analysis

Supplementary Table S2: List of rodent-borne zoonotic diseases

1 Bacterial diseases

#	Disease	Causal agent	Rodent role	References
1	Bartonellosis	Bartonella spp. such as *B. doshiae, B. elizabethae, B. grahamii, B. rochalimae, B. tribocorum, B. vinsonii subsp. Arupensis*, and *B. washoensis*	Reservoir	[1-5]
2	Campylobacteriosis	*Campylobacter* spp., such as *C. coli, C. fetus, C. hyointestinalis,* and *C. jejuni*	Carrier	[2,3,6-9]
3	Corynobacteriosis */Non-diphtheritic Corynebacterium*	*Corynebacterium* spp.	Reservoir	[3,6]
4	E. coli enteritis	*Escherichia coli* 0157/VTEC	Carrier	[2,3,9]
5	Ehrlichiosis	*Ehrlichia* spp. such as *E. chaffeensis, E. equi, E. ewingii, E. morulae, E. muris, E. phagocytophilum, E. risticii,* and *E. sennetsu*	Reservoir	[1,10-12]
6	Leptospirosis	*Leptospira interrogans*	Carrier	[1-3]
7	Listeriosis	*Listeria* spp. such as *L. monocytogenes* and *L. ivanovii*	Carrier	[2,3,13]
8	Lyme disease	*Borrelia burgdorferi*	Reservoir	[1-3]
9	Pasteurellosis	*Pasteurella* spp.	Reservoir	[3,14]
10	Plague	*Yersinia pestis*	Carrier	[1-3]
11	Rickettsial Pox	*Rickettsia akari*	Reservoir	[1-3]
12	Q-Fever	*Coxiella burnetii*	Carrier	[2,3]
13	Rat-bite fever	*Spirillum minus and S. moniliformis*	Carrier	[2,3]
14	Relapsing fever (Tick-borne/Lice borne)	*Borrelia* spp. such as *B. duttoni, B. hermsii, B. parkeri, B. recurrentis,* and *B. turicatae*	Reservoir	[1-3,15]
15	Salmonellosis	*Salmonella spp.* such as *S. enterica* and *S. enteritidis*	Reservoir	[2,3,6,9]
16	Japanese spotted fever	*Rickettsia japonica*	Reservoir	[1]
17	Mediterranean spotted fever /Boutonneuse	*Rickettsia conorii*	Reservoir	[3]
	Queensland spotted fever/ tick typhus	*Rickettsia australis*	Reservoir	[2]
18	Rocky mountain spotted fever	*Rickettsia rickettsi*	Reservoir	[1-3]
19	Tuberculosis	*Mycobacterium microti, a member of Mycobacterium tuberculosis* complex	Reservoir	[3,16-18]
20	Tularemia	*Francisella tularensis*	Carrier	[1-3]
21	Murine typhus /Endemic typhus	*Rickettsia typhi*	Reservoir	[1-3]
22	Scrub Typhus	*Orientia tsutsugamushi*	Reservoir	[1,2]
23	Siberian Typhus	*Rickettsia sibirica*	Reservoir	[1]
24	Sylvatic Typhus /Epidemic thymus	*Rickettsia prowazekii*	Reservoir	[1-3]

25	Yersiniosis	*Yersinia pseudotuberculosis, Y. enterocolitis*	Reservoir	[1,3]

2 Fungal diseases

#	Disease	Causal agent	Rodent role	References
1	Dermatophytosis	Dermatophytes fungus	Carrier	[1]
2	Histoplasmosis	*Histoplasma capsulatum*	Reservoir	[1,19]
3	Penicilliosis	*Penicillium marneffei*	Reservoir	[1,20]

3 Parasitic diseases

#	Disease	Causal agent (Type, name)	Rodent role	References
1	Moniliformiasis	Acanthocephala, *Moniliformis moniliformis*		[3]
2	Echinococcosis	Cestode, *Echinococcus* spp., such as *E. granulosus, E. multilocularis, E. oligarthrus,* and *E. vogeli*	Reservoir	[1-3,21,22]
3	Hymenolepiasis	Cestode, *Hymenolepis diminuta, H. nana,* and *H. microstoma*	Reservoir	[1-3,23]
4	Taeniasis	Cestode, *Taenia* spp., such as *T. brauni, T.crassiceps, T. glomeratus, T. hydatigena, T. martis, T. pisiformis, T. serialis, T. taeniaeformis, Versteria mustelae*	Reservoir	[2,3,24,25]
5	Aelurostrongylosis	Nematode, *Aelurostrongylus abstrusus*	Reservoir	[2]
6	Angiostrongyliasis	Nematode, *Angiostrongylus cantonensis*	Reservoir	[1-3]
7	Baylisascariasis	Nematode, *Baylisascaris procyonis*	Reservoir	[1-3]
8	Capilariasis	Nematode, *Capilaria hepatica*	Reservoir	[1-3]
9	Gongylonemiasis	Nematode, *Gongylonema pulchrum*		[3,26]
10	Toxascariasis	Nematode, *Toxascaris leonina*	Reservoir	[2,3]
11	Trichinellosis	Nematode, *Trichinella* spp., such as *Trichinella spiralis*	Reservoir	[2,3,27]
12	Trichuriasis	Nematode, *Trichuris spp.*		[3]
13	Alariasis	Trematode, *Alaria spp.,* such as *A. alata* and *A. Americana*	Reservoir	[2,3,28,29]
14	Brachylaimiasis	Trematode, *Brachylaima cribbi*	Reservoir	[2,3]
15	Echinostomiasis	Trematode, *Echinosoma spp.* such as *E. echinatum, E. japonicus, E. perfoliatus, E. cinetorchis, E. Hortense, E. ilocanum, E. revolutum, Isthmiophora melis*	Reservoir	[1-3,30-32]
16	Fascioliasis	Trematode, *Fasciola hepatica, F. gigantica*	Reservoir	[1-3]
17	Plagiorchiasis	Trematode, *Plagiorchis muris, P. hilippinensis, P. javanensis*		[3]
18	Schistosomiasis	Trematode, *Schistosoma mansoni*	Reservoir	[1-3]
19	Amoebic dysentery	Protozoa, *Entamoeba histolytica*	Reservoir	[2]
20	Anaplasmosis/ Human granulocytic analplasmosis	Protozoa, *Anaplasma phagocytophilum*	Reservoir	[1-3]
21	Babesiosis	Protozoa, *Babesia spp.* such as *Babesia microti*	Reservoir	[2,3,33]
22	Cryptosporidiasis	Protozoa, *Cryptosporidium spp.* (*Cryptosporidium parvum, Cryptosporidium hominis, Cryptosporidium meleagridis, Cryptosporidium muris)*	Reservoir	[1-3,34,35]

23	Giardiasis	Protozoa, *Giardia lamblia*	Reservoir	[1-3]
24	Leishmaniasis (cutaneous, mucocutaneous, and visceral)	Protozoa, *Leishmania braziliensis, L. donovani, L. infantum, L. major, and L. tropica*	Reservoir	[1-3]
25	Neosporosis	Protozoa, *Neospora caninum*	Reservoir	[2,3]
26	Toxoplasmosis	Protozoa, *Toxoplasma gondii*	Reservoir	[1-3]
27	Trypanosomiasis	Protozoa, *Trypanosoma brucei* and T. *cruzi*	Reservoir	[1-3]

4 Virus

#	Disease	Causal agent	Rodent role	References
1	Argentine/Junin/Mapucho hemorrhagic fever	*South American arenavirus,* Arenaviridae	Reservoir	[1-3,36]
2	Bolivian hemorrhagic fever	*Machupo virus,* Arenaviridae	Reservoir	[1,3,36]
3	Hemorrhagic fever Brazilian	*Sabia virus,* Arenaviridae	Reservoir	[3,36]
4	Lujo Hemorrhagic fever	*Lujovirus,* Arenaviridae	Reservoir	[3,36]
5	Venezuelan hemorrhagic fever	*Guanaritovirus,* Arenaviridae	Reservoir	[1,3,36]
6	Lassa fever	Lassa virus (*LASV),* Arenaviridae	Reservoir	[1-3,36]
7	Lymphocytic choriomeningitis	Lymphocytic choriomeningitis *(LCMV),* Arenaviridae	Reservoir	[1-3,36]
8	North American arenavirus infection	*North American arenavirus,* Arenaviridae	Reservoir	[2,36]
9	Whitewater Arroyo virus infection	Whitewater Arroyo virus (*WWAV*), Arenaviridae	Reservoir	[1,36]
10	Borna disease	*Borna virus,* Bornaviridae	Reservoir	[2,3]
11	Hantavirus pulmonary syndrome	*Hantavirus,* Hantaviridae	Carrier	[1-3]
12	Hemorrhagic fever with renal syndrome	*Hantavirus,* Hantaviridae	Carrier	[2,3]
13	Nephropathia epidemica	*Hantavirus,* Hantaviridae	Carrier	[2]
14	Ebola	*Ebola virus,* Filoviridae	Reservoir?	[1]
15	Apoi virus disease	*Apoi virus,* Flaviviridae	Unknown	[2,3]
16	Tick-borne encephalitis	*TBE virus,* Flaviviridae	Reservoir	[1-3]
17	Omsk hemorrhagic fever	*OHF virus,* Flaviviridae	Reservoir	[2,3]
18	Kyasanur forest disease	*Kyasanur forest virus,* Flaviviridae	Reservoir	[2,3]
19	Powassan encephalitis	*Powassan virus,* Flaviviridae	Reservoir	[1-3]
20	West Nile fever	*West Nile virus,* Flaviviridae	Reservoir	[1]
21	Hepatitis E	*Orthohepevirus A,* Hepeviridae	Reservoir	[1-3]
22	Crimean-Congo hemorrhagic fever	*CCHF virus,* Nairoviridae	Reservoir	[1-3]
23	Encephalitis California	*California Encephalitis virus,* Peribunyaviridae	Carrier	[1]
24	Rift Valley fever	*Phlebovirus,* Phenuiviridae	Carrier	[1,3]
25	Cow Pox	*Cowpox/Vacciniavirus,* Poxviridae	Reservoir	[1-3]
26	Monkey Pox	*Monkeypox virus,* Poxviridae	Reservoir	[1,37]
27	Colorado tick fever	*Caltivirus,* Reoviridae	Reservoir	[1-3]

28	Rabies	*Rabies virus,* Rhabdoviridae	Reservoir	[1,3,38]
29	Chikungunya	*Chikungunya virus,* Togaviridae	Reservoir	[1,39]
30	Eastern Equine Encephalitis	*EEE virus,* Togaviridae	Reservoir	[1]
31	Venezuelan Equine Encephalitis	*VEE virus,* Togaviridae	Reservoir	[1-3]
32	Western Equine Encephalitis	*WEE virus,* Togaviridae	Reservoir	[2,3]
33	Mayaro	*Mayarovirus,* Togaviridae	Reservoir	[1]

5 References

1. Han, B.A.; Schmidt, J.P.; Bowden, S.E.; Drake, J.M. Rodent reservoirs of future zoonotic diseases. *Proceedings of the National Academy of Sciences* **2015**, *112*, 7039, doi:10.1073/pnas.1501598112.
2. Meerburg, B.G.; Singleton, G.R.; Kijlstra, A. Rodent-borne diseases and their risks for public health. *Crit Rev Microbiol* **2009**, *35*, 221-270, doi:10.1080/10408410902989837.
3. Rabiee, M.H.; Mahmoudi, A.; Siahsarvie, R.; Kryštufek, B.; Mostafavi, E. Rodent-borne diseases and their public health importance in Iran. *PLoS Negl Trop Dis* **2018**, *12*, e0006256, doi:10.1371/journal.pntd.0006256.
4. Favacho, A.R.d.M.; Andrade, M.N.; de Oliveira, R.C.; Bonvicino, C.R.; D'Andrea, P.S.; de Lemos, E.R.S. Zoonotic Bartonella species in wild rodents in the state of Mato Grosso do Sul, Brazil. *Microbes and Infection* **2015**, *17*, 889-892, doi:https://doi.org/10.1016/j.micinf.2015.08.014.
5. Gonçalves, L.R.; Favacho, A.R.d.M.; Roque, A.L.R.; Mendes, N.S.; Fidelis Junior, O.L.; Benevenute, J.L.; Herrera, H.M.; Andrea, P.S.; de Lemos, E.R.S.; Machado, R.Z., et al. Association of Bartonella Species with Wild and Synanthropic Rodents in Different Brazilian Biomes. *Applied and Environmental Microbiology* **2016**, *82*, 7154, doi:10.1128/AEM.02447-16.
6. Meerburg, B.G.; Kijlstra, A. Role of rodents in transmission of Salmonella and Campylobacter. *Journal of the Science of Food and Agriculture* **2007**, *87*, 2774-2781, doi:10.1002/jsfa.3004.
7. Meerburg, B.G.; Jacobs-Reitsma, W.F.; Wagenaar, J.A.; Kijlstra, A. Presence of Salmonella and Campylobacter spp. in wild small mammals on organic farms. *Applied and environmental microbiology* **2006**, *72*, 960-962, doi:10.1128/AEM.72.1.960-962.2006.
8. Edmonds, P.; Patton, C.M.; Griffin, P.M.; Barrett, T.J.; Schmid, G.P.; Baker, C.N.; Lambert, M.A.; Brenner, D.J. Campylobacter hyointestinalis associated with human gastrointestinal disease in the United States. *J Clin Microbiol* **1987**, *25*, 685-691.
9. Nkogwe, C.; Raletobana, J.; Stewart-Johnson, A.; Suepaul, S.; Adesiyun, A. Frequency of Detection of Escherichia coli, Salmonella spp., and Campylobacter spp. in the Faeces of Wild Rats (Rattus spp.) in Trinidad and Tobago. *Vet Med Int* **2011**, *2011*, 686923-686923, doi:10.4061/2011/686923.
10. Thomas, R.J.; Dumler, J.S.; Carlyon, J.A. Current management of human granulocytic anaplasmosis, human monocytic ehrlichiosis and Ehrlichia ewingii ehrlichiosis. *Expert Rev Anti Infect Ther* **2009**, *7*, 709-722, doi:10.1586/eri.09.44.
11. Kawahara, M.; Ito, T.; Suto, C.; Shibata, S.; Rikihisa, Y.; Hata, K.; Hirai, K. Comparison of Ehrlichia muris Strains Isolated from Wild Mice and Ticks and Serologic Survey of Humans and Animals with E. muris as Antigen. *J Clin Microbiol* **1999**, *37*, 1123, doi:10.1128/JCM.37.4.1123-1129.1999.
12. Tominello, T.R.; Oliveira, E.R.A.; Hussain, S.S.; Elfert, A.; Wells, J.; Golden, B.; Ismail, N. Emerging Roles of Autophagy and Inflammasome in Ehrlichiosis. *Front Immunol* **2019**, *10*, 1011, doi:10.3389/fimmu.2019.01011.
13. Wang, Y.; Lu, L.; Lan, R.; Salazar, J.K.; Liu, J.; Xu, J.; Ye, C. Isolation and characterization of Listeria species from rodents in natural environments in China. *Emerg Microbes Infect* **2017**, *6*, e44, doi:10.1038/emi.2017.28.

14. Scharmann, W.; Heller, A. Survival and transmissibility of Pasteurella pneumotropica. *Lab Anim* **2001**, *35*, 163-166, doi:10.1258/0023677011911543.
15. Dworkin, M.S.; Schwan, T.G.; Anderson, D.E., Jr.; Borchardt, S.M. Tick-borne relapsing fever. *Infect Dis Clin North Am* **2008**, *22*, 449-viii, doi:10.1016/j.idc.2008.03.006.
16. van Soolingen, D.; van der Zanden, A.G.; de Haas, P.E.; Noordhoek, G.T.; Kiers, A.; Foudraine, N.A.; Portaels, F.; Kolk, A.H.; Kremer, K.; van Embden, J.D. Diagnosis of Mycobacterium microti infections among humans by using novel genetic markers. *J Clin Microbiol* **1998**, *36*, 1840-1845, doi:10.1128/JCM.36.7.1840-1845.1998.
17. Behr, M.A.; Gagneux, S. 24 - The Rise and Fall of the Mycobacterium tuberculosis Complex. In *Genetics and Evolution of Infectious Disease*, Tibayrenc, M., Ed. Elsevier: London, 2011; https://doi.org/10.1016/B978-0-12-384890-1.00024-8pp. 651-667.
18. Moradi, E.; Mosavari, N.; Tebianian, M.; Tadayon, K.; Ghaderi, R.; Soleymani Babadi, K.; Mohammad Taheri, M.; Dashtipour, S.; Loni, R.; Moradi Garavand, M., et al. Pest rodents as the essential elements of Mycobacterium bovis controlling programs. *International Journal of Mycobacteriology* **2015**, *4*, 137, doi:https://doi.org/10.1016/j.ijmyco.2014.09.007.
19. Emmons, C.W.; Ashburn, L.L. Histoplasmosis in Wild Rats: Occurrence and Histopathology. *Public Health Reports (1896-1970)* **1948**, *63*, 1416-1422, doi:10.2307/4586744.
20. Cao, C.; Liang, L.; Wang, W.; Luo, H.; Huang, S.; Liu, D.; Xu, J.; Henk, D.A.; Fisher, M.C. Common reservoirs for Penicillium marneffei infection in humans and rodents, China. *Emerg Infect Dis* **2011**, *17*, 209-214, doi:10.3201/eid1702.100718.
21. da Silva, A.M. Human echinococcosis: a neglected disease. *Gastroenterol Res Pract* **2010**, *2010*, 583297, doi:10.1155/2010/583297.
22. Tappe, D.; Stich, A.; Frosch, M. Emergence of polycystic neotropical echinococcosis. *Emerg Infect Dis* **2008**, *14*, 292-297, doi:10.3201/eid1402.070742.
23. Macnish, M.G.; Ryan, U.M.; Behnke, J.M.; Thompson, R.C. Detection of the rodent tapeworm Rodentolepis (=Hymenolepis) microstoma in humans. A new zoonosis? *Int J Parasitol* **2003**, *33*, 1079-1085, doi:10.1016/s0020-7519(03)00137-1.
24. Deplazes, P.; Eichenberger, R.M.; Grimm, F. Wildlife-transmitted Taenia and Versteria cysticercosis and coenurosis in humans and other primates. *Int J Parasitol Parasites Wildl* **2019**, *9*, 342-358, doi:10.1016/j.ijppaw.2019.03.013.
25. Spickler, A.R. Taeniasis, Cysticercosis, and coenurosis. In *Date of Factsheet*, 2020.
26. da Costa Cordeiro, H.; de Vasconcelos Melo, F.T.; Giese, E.G.; Santos, J.N.D. Gongylonema Parasites of Rodents: A Key to Species and New Data on Gongylonema neoplasticum. *J Parasitol* **2018**, *104*, 51-59, doi:10.1645/17-3.
27. Pozio, E. World distribution of Trichinella spp. infections in animals and humans. *Vet Parasitol* **2007**, *149*, 3-21, doi:10.1016/j.vetpar.2007.07.002.
28. Möhl, K.; Grosse, K.; Hamedy, A.; Wüste, T.; Kabelitz, P.; Lücker, E. Biology of Alaria spp. and human exposition risk to Alaria mesocercariae-a review. *Parasitol Res* **2009**, *105*, 1-15, doi:10.1007/s00436-009-1444-7.
29. Wasiluk, A. Alaria alata infection - threating yet rarely detected trematodiasis. *Journal of Laboratory Diagnostics* **2013**.
30. Hildebrand, J.; Adamczyk, M.; Laskowski, Z.; Zaleśny, G. Host-dependent morphology of Isthmiophora melis (Schrank, 1788) Luhe, 1909 (Digenea, Echinostomatinae)--morphological variation vs. molecular stability. *Parasit Vectors* **2015**, *8*, 481-481, doi:10.1186/s13071-015-1095-8.
31. Toledo, R.; Esteban, J.G. An update on human echinostomiasis. *Trans R Soc Trop Med Hyg* **2016**, *110*, 37-45, doi:10.1093/trstmh/trv099.
32. Gillespie, S.H.; Pearson, R.D. Principles and practice of clinical parasitology. **2010**.
33. Bloch, E.M.; Kumar, S.; Krause, P.J. Persistence of Babesia microti Infection in Humans. *Pathogens* **2019**, *8*, 102, doi:10.3390/pathogens8030102.
34. García-Livia, K.; Martín-Alonso, A.; Foronda, P. Diversity of Cryptosporidium spp. in wild rodents from the Canary Islands, Spain. *Parasit Vectors* **2020**, *13*, 445, doi:10.1186/s13071-020-04330-9.
35. Lv, C.; Zhang, L.; Wang, R.; Jian, F.; Zhang, S.; Ning, C.; Wang, H.; Feng, C.; Wang, X.; Ren, X., et al. Cryptosporidium spp. in Wild, Laboratory, and Pet Rodents in China: Prevalence and Molecular Characterization. *Applied and Environmental Microbiology* **2009**, *75*, 7692, doi:10.1128/AEM.01386-09.

36. Gravinatti, M.L.; Barbosa, C.M.; Soares, R.M.; Gregori, F. Synanthropic rodents as virus reservoirs and transmitters. *Rev Soc Bras Med Trop* **2020**, *53*, e20190486, doi:10.1590/0037-8682-0486-2019.
37. Buller, R.M.L. 170 - Poxviruses. In *Infectious Diseases (Fourth Edition)*, Cohen, J., Powderly, W.G., Opal, S.M., Eds. Elsevier: 2017; https://doi.org/10.1016/B978-0-7020-6285-8.00170-2pp. 1452-1457.e1451.
38. Fitzpatrick, J.L.; Dyer, J.L.; Blanton, J.D.; Kuzmin, I.V.; Rupprecht, C.E. Rabies in rodents and lagomorphs in the United States, 1995-2010. *J Am Vet Med Assoc* **2014**, *245*, 333-337, doi:10.2460/javma.245.3.333.
39. Vourc'h, G.; Halos, L.; Desvars, A.; Boué, F.; Pascal, M.; Lecollinet, S.; Zientara, S.; Duval, T.; Nzonza, A.; Brémont, M. Chikungunya antibodies detected in non-human primates and rats in three Indian Ocean islands after the 2006 ChikV outbreak. *Vet Res* **2014**, *45*, 52-52, doi:10.1186/1297-9716-45-52.
