# Supplementary material for: Rodent-Related Zoonotic Pathogens at the Human–Animal–Environment Interface in Qatar: A Systematic Review and Meta-Analysis
Source: Int J Environ Res Public Health. 2021 May 31;18(11):5928. doi: 10.3390/ijerph18115928 (PMC8198466; doi:10.3390/ijerph18115928)
Supplement: Supplementary file 1 [file ijerph-18-05928-s001.zip › Supplementary Table S3.pdf]

Rodent related zoonotic pathogens at the human-animal-environmental interface in Qatar: a systematic review and meta-analysis

Supplementary Table S3: The extracted data from the included articles

Authors and References	Study type and period	Study subjects	Pathogens name	Sample details (Total sample/Total positive)					Additional information
				Qatari	African	South-East Asian	West Asian	Grand Total	
Abbas *et al.*, 2014 [1]	Cross-sectional, January 2009 to December 2010	Human: liver and stool of hepatic abscess patient	*Escherichia coli*					67/56	
			Entamoeba histolytica					67/11	
Abdelmaguid *et al.*, 2019 [2]	January 2016 to January 2017	Human: CSF of acute meningitis patients	*Escherichia coli*					50/2	
Abu-Madi *et al.*, 2016a [3]		Non-human: chicken carcass in the retail market	*Campylobacter jejuni*					400/188	*Campylobacter* contamination is associated with market management systems, especially during preparation, storage, and display.
			Campylobacter coli					400/1	
Abu-Madi *et al.*, 2017 [4]	Cross-sectional, 2012-2014	Human: stool of newly arrived immigrants	*Entamoeba histolytica*		89/1	646/87		735/12	
			Giardia duodenalis		89/18	646/105		735/105	
Abu-Madi *et al.*, 2011a [5]	Cross-sectional, June 1998 to April 1999	Non-human: stool of *Rattus norvegicus*	*Hymenolepis diminuta*					136/24	Adult rats had an overall higher prevalence than juvenile rats
Abu-Madi *et al.*, 2005 [6]	Cross-sectional, February -April 2002 and 2003	Non-human: stool of *Rattus norvegicus*	*Hymenolepis diminuta*					179/64	The prevalence increases with age
Abu-Madi *et al.*, 2007 [7]	Cross-sectional, January to October 2005	Non-human: stray cat stool	*Taenia* sp.					824/88	
			Toxascaris leonina					824/6	

Authors and References	Study type and period	Study subjects	Pathogens name	Sample details (Total sample/Total positive)					Additional information
				Qatari	African	South-East Asian	West Asian	Grand Total	
Abu-Madi *et al.*, 2008a [8]	Cross-sectional, January-October, 2006	Non-human: stray cat stool	*Taenia taeniaeformis*					488/370	The cestode prevalence is higher among adult cats than youngs.
Abu-Madi *et al.*, 2010a [9]	Cross-sectional, 2006-07	Non-human: feral cat stool (n=658)	*Taenia taeniaeformis*					658/485	
			Toxascaris leonina					658/1	
Abu-Madi *et al.*, 2011b [10]	Cross-sectional, June-September 2009	Human: stool of residents	*Giardia duodenalis*		424/11	1114/23		1538/34	*Schistosoma* was in long-term resident workers, whereas other parasites were more frequent in newly arrived persons.
			Trichuris trichiura		424/11	1114/43		1538/54	
			Entamoeba histolytica/dispar					1538/12	
			Hymenolepis nana					1538/15	
			Taenia spp.					1538/8	
			Schistosoma spp.					1538/2	
Abu-Madi *et al.*, 2016b [11]	Retrospective, 2005-2014	Human: stool of the residents	*Giardia duodenalis*	9357/67	5354/90		4240/42	29286/431	Male had a higher prevalence than females, and the Asian immigrants were more infected with *Giardia* and amoeba than the others and younger than adults.
			Cryptosporidium spp.					29286/15	
			Entamoeba histolytica/dispar	9357/4	5354/15		4240/5	29286/62	
Abu-Madi *et al.*, 2016c [12]	Cross-sectional, 2012-2014	Human: stool of the residents	*Trichuris trichiura*		261/2	2225/32		2486/34	Parasite prevalence depends on the immigrants' origin. *Trichuris* and *Giardia* were more prevalent among the Asians than others, whereas other parasites were more among the African immigrants.
			Taenia spp.		261/1	2225/1		2486/2	
			Hymenolepis nana		261/2	2225/8		2486/10	
			Entamoeba histolytica/dispar		261/3	2225/20		2486/23	
			Giardia duodenalis		261/3	2225/56		2486/59	
	Retrospective, 2005-2014	Human: stool of the residents	*Trichuris trichiura*	9357/0	5354/3		4240/0	29286/97	*Schistosoma* spp., and *Taenia* spp. were rare infections. The
			Hymenolepis nana	9357/1	5354/10		4240/5	29286/43	

Authors and References	Study type and period	Study subjects	Pathogens name	Sample details (Total sample/Total positive)					Additional information
				Qatari	African	South-East Asian	West Asian	Grand Total	
Abu-Madi *et al.*, 2016d [13]			*Schistosoma* spp.					29286/18	prevalence was seven times higher in males than females.
			Taenia spp.					29286/9	
Abu-Madi *et al.*, 2008b [14]	Retrospective, 2005-2007	Human: hospital bases serosurvey	*Toxoplasma gondii*		304/45	511/153	810/280	1625/484	Overall, the prevalence increases with age after >45 years (41%).
Abu-Madi *et al.*, 2010b [15]	Longitudinal, 2005-2008	Human: TORCH* patients' blood serum	*Toxoplasma gondii*		309/129	608/182	901/247	1857/572	About 22.9% of the infants were seropositive indicates that toxoplasmosis has congenital transmission; the seroprevalence increases with age.
Abu-Madi *et al.*, 2010c [16]	Retrospective, 2005-2008	Human: stool of long-term residents	*Giardia duodenalis*	3310/34	1299/16	3214/34	1385/95	9208/179	Children were more susceptible to *G. duodenalis*.
			Entamoeba histolytica/diapar	3310/4	1299/7	3214/15	1385/1	9208/27	
			Trichuris trichiura	3310/0	1299/1	3214/44	1385/0	9208/45	
			Hymenolepis nana	3310/0	1299/3	3214/5	1385/1	9208/9	
Abu-Madi *et al.*, 2013 [17]	Retrospective, 2005 to 2011	Human: stool of long-term residents	*Giardia duodenalis*					18563/316	
			Trichuris trichiura	6413/0	2998/3		2650/0	18563/84	
			Hymenolepis nana	6413/0	2998/8		2650/16	18563/43	
Abu-Madi *et al.*, 2008c [18]	Cross-sectional, 2005-2006	Human: stool of food handlers and housemaids	*Trichuris trichiura*		258/12	1479/122		1737/134	Females were more infected than males with these parasites. *Giardia duodenalis* is a synonym of *Giardia lamblia*
			Entamoeba histolytica/dispar		258/2	1479/23		1737/25	
			Giardia duodenalis		258/13	1479/74		1737/87	
Ahmed *et al.*, 2016 [19]	Prospective, November 2012 to October 2013	Human: ICU patients' blood, respiratory, urine, and body fluids	*Escherichia coli* (ESBL producing)					629/38	*E. coli* was one of the critical ESBL producing enterobacters isolated from the ICU patients samples.

Authors and References	Study type and period	Study subjects	Pathogens name	Sample details (Total sample/Total positive)					Additional information
				Qatari	African	South-East Asian	West Asian	Grand Total	
Ahmedullah *et al.*, 2018 [20]	Retrospective, 2005-2012	Human: blood and stool of adult patients with typhoid fever	*Salmonella enterica* serotype Typhi	-/12		-/326		-/354	Out of the 354 patients, 83.6% were males. Most of the patients (96.9%) traveled to the Indian subcontinent (93%) before they got infected.
Al Alousi *et al.*, 2012 [21]	Retrospective, January 2002-December 2008	Human: sputum of pulmonary TB patients	*Mycobacterium tuberculosis*					-/687	A sputum culture can be a sample of choice for acid-fast bacilli detection.
Al-Aani *et al.*, 2014 [22]	Retrospective, January 2000 to December 2013	Human	*Echinococcus granulosus*	-/3				-/32	Hydatid disease was confirmed patients mainly were (90.6%) non-Qatari, 25-34 years old, and predominantly (63%) male.
Al Jama, 2012 [23]	Retrospective, January 1998 to December 2007	Human: surgical wound	*Escherichia coli*					107/18	*E. coli* can be a significant cause of infection after lower segment cesarean section
Al Marri *et al.*, 2006 [24]	Retrospective, 1993-2000	Human: expatriate who seek Qatari residence	*Mycobacterium tuberculosis*					32134/200	
Al Marri et al., 2000 [25]	Retrospective, 1980-1988	Human: women with breast TB	*Mycobacterium tuberculosis*					300/2	Breast TB cases were more among the young multiparous women.
Al Marri et al., 2002 [26]	Retrospective, January 1992-December 1996	Human: different pediatric TB patients	*Mycobacterium tuberculosis*	-/34				-/66	
Al Marri, 2012 [27]	Retrospective, January-December 1998	Human: pulmonary TB patients	*Mycobacterium tuberculosis*	-/59				-/306	False tuberculin negative results can be a cause to spread TB in Qatar.
Al Soub, *et al.*, 2016 [28]	Case report	Human: blood from a senior male American citizen	*Babesia* sp.					-/1	The first human babesiosis case in Qatar.

Authors and References	Study type and period	Study subjects	Pathogens name	Sample details (Total sample/Total positive)					Additional information
				Qatari	African	South-East Asian	West Asian	Grand Total	
Al-Aani *et al.*, 2009 [29]	Case report	Human: meningitis	*Salmonella paratyphi*					-/1	The patient was coinfected with *Salmonella* in the meninges along with Hepatitis B
Al-Absi *et al.*, 2018 [30]	Cross-sectional, June 2013 to June 2016	Human: routine serosurvey of the blood of donors	Hepatitis E virus					1049/156	Hepatitis E seroprevalence is higher in males than females and 25 to 45 age among the blood donors.
Al-Baker, et al., 2018 [31]	Retrospective, March 2007 to February 2011	Human: uveitis patients	*Mycobacterium tuberculosis*					310/45	
			Toxoplasma sp.					310/16	
Al-Dahshan *et al.*, 2019 [32]	Cross-sectional, 2017	Human: stool and blood from pediatric (infant) salmonellosis cases	*Salmonella* (Type B, C, D, and E)	-/15	-/15	-/18		-/89	The identified *Salmonella* were type B 50%, C 14%, D 18%, and E 1%. Formula food can be the source of *Salmonella* infection among infants in Qatar.
Alho *et al.*, 2017 [33]	March to July 2016	Non-human: pet dog stool	*Anaplasma platys*					64/1	Among the dogs, 18.8% were positive for at least one parasite, whereas 20.6% (n=7) of cats were positive for at least one parasite. Co-infection with more than one parasite was also detected.
			Babesia gibsoni					64/1	
			Babesia vogeli					64/2	
		Non-human: pet cat stool	*Babesia vogeli*					34/1	
Ali *et al.*, 2019 [34]	Case report	Human: tuberculous pancreatitis	*Mycobacterium tuberculosis*					-/1	The first case of pancreatic TB was recorded in an Indian migrant.
Al-Jedah and Robinson, 2002 [35]	Cross-sectional	Mixed fresh fruit Juice from local restaurants	*Escherichia coli*					10/6	The isolated *E. coli* total bacterial counts were above the Gulf countries' standard.

Authors and References	Study type and period	Study subjects	Pathogens name	Sample details (Total sample/Total positive)					Additional information
				Qatari	African	South-East Asian	West Asian	Grand Total	
Al-Khal et al., 2005 [36]	Retrospective, October 2001-2003	Human: garments workers, tuberculin test	*Mycobacterium tuberculosis*			2774/1193		2774/1193	The positive garment workers were mostly Indians (12.9%), Bangladeshi (38%), Nepali (46.6%), Sri Lankan (2%), and Pakistani (0.4%)
Al-Marri, 2001a [37]	Retrospective, 1983-1996	Human: pediatric tuberculosis	*Mycobacterium tuberculosis*					-/144	The annual caseload was 5-10% from 1983 to 1996. The policy of BCG vaccination would be helpful for TB control among children.
Al-Marri, 2001b [38]	Retrospective, January 1996-December 1998	Human: pulmonary TB	*Mycobacterium tuberculosis*	-/65	-/17	-/275	-/35	-/406	
Al-Marri and Kirkpatrick, 1999 [39]	Retrospective, 1983-1996	Human: pediatric tuberculous patients	*Mycobacterium tuberculosis*					-/144	
Al-Mulla *et al.*, 2014 [40]	Retrospective, 2004-2011	Human: blood from pediatric hematology/oncology cases	*Escherichia coli*					185/7	
			Salmonella spp.					185/3	
Al-Nesf *et al.*, 2019 [41]	Retrospective, January 1997 to December 2007	Human: patient with hemoptysis	*Mycobacterium tuberculosis*					102/5	
Al-Shaer *et al.*, 2017 [42]	Retrospective cohort, December 2012-November 2014	Human: sputum from pulmonary TB	*Mycobacterium tuberculosis*					-/148	Tuberculous patients were mainly male (85.8%) and from Asia (87%).

Authors and References	Study type and period	Study subjects	Pathogens name	Sample details (Total sample/Total positive)					Additional information
				Qatari	African	South-East Asian	West Asian	Grand Total	
Al-Shaer *et al.*, 2018 [43]	Retrospective cohort, December 2012 to December 2015	Human: sputum from pulmonary TB	*Mycobacterium tuberculosis*					-/103	
Alsoub and Al Alousi, 2001 [44]	Retrospective, January 1992-December 1998	Human: miliary nodules by radiograph	*Mycobacterium tuberculosis*	-/3		-/26		-/32	Miliary TB is prevalent among the Indian origin patients
Al-Thani *et al.*, 2013 [45]	Cross-sectional, June 2009-November 2009	Human: stool from acute gastroenteritis patients	*Campylobacter* sp.					288/3	These bacterial infections were associated with viral infections such as norovirus, rotavirus, adenovirus, and astrovirus.
			Escherichia coli					288/6	
			Salmonella sp.					288/23	
Ben Abid and Al Soub, 2018 [46]	Case report	Human: Indian origin resident	*Mycobacterium tuberculosis*					-/1	The woman got tuberculous mastitis, who had a family history of TB
Boughattas *et al.*, 2017a [47]	Cross-sectional, March 2015 to March 2016	Human: stool from pediatric diarrheal patients	*Giardia duodenalis*					580/10	Generally, the prevalence was highest among Qatari, older children (5+ years), female children, and during the winter. *G. duodenalis* was highest among Asian children.
			Cryptosporidium parvum					580/85	
			Cryptosporidium hominis					580/5	
			Cryptosporidium meleagridis					580/2	
			Entamoeba histolytica					580/1	
Boughattas *et al.*, 2017b [48]		Non-human: feral cats' blood serum	*Toxoplasma gondii*					495/406	Seroprevalence was higher in summer (84.5%) than winter (72.3%).
Boughattas *et al.*, 2019 [49]	Cross-sectional survey, 2012–2014	Human: newly arrived immigrants' stool	*Cryptosporidium parvum*<br>*Cryptosporidium hominis*<br>*Cryptosporidium meleagridis*		52/34	787/37		839/38	The positive cases were newly arrived residents. Poor education and pit latrines users (in the country of origin) were significantly highly positive.

Authors and References	Study type and period	Study subjects	Pathogens name	Sample details (Total sample/Total positive)					Additional information
				Qatari	African	South-East Asian	West Asian	Grand Total	
Chang *et al*., 2016 [50]	Repeat cross-sectional, 2011	Human: stool	*Salmonella enterica*					-/69	There is a zoonotic transmission of *S. enterica* between humans and animals in Qatar.
		Non-human: Cattle feces and bedding						-/8	
		Non-human: Camel carcass						-/1	
Derbala *et al*., 2015 [51]	Retrospective, January 2006 to April 2014	Human: Serology	*Schistosoma mansoni*					383/128	Anti-schistosomal antibody was detected among the adults who took treatment for Hepatitis C.
Dousa *et al*., 2019 [52]	Retrospective, January 2011-January 2015	Human: Sputum culture and stain from pulmonary TB patients	*Mycobacterium tuberculosis*					-/134	Persons with diabetes mellitus have an increased risk of getting TB
Dubey *et al*., 2010 [53]		Non-human: Sand cat, wild cat, and big cat (Chittah) tissue and serum	*Toxoplasma gondii*					27/21	
Ehlayel et al., 2013. [54]	Retrospective, 1998-2012	Human: Children with primary immunodeficiency	*Salmonella* spp.					131/16	*Salmonella* recurrent infection is one of the common causes of primary immunodeficiency
El-Nemr *et al*., 2019 [55]	Cross-sectional	Human and Non-human: Fresh product, hand swab, environment, and surface sample from the wholesale market	*Corynebacterium* spp.					105/4	The research showed that these two bacteria could spread from the wholesale market to entire Qatar.
			Escherichia/Shigella/Enterobacter spp.					105/8	
Eltai *et al*., 2020a [56]	Cross-sectional, December 2016 to July 2017	Non-human: sheep feces	*Escherichia coli*					171/144	

Authors and References	Study type and period	Study subjects	Pathogens name	Sample details (Total sample/Total positive)					Additional information
				Qatari	African	South-East Asian	West Asian	Grand Total	
Eltai *et al.*, 2018a [57]	Cross-sectional, August 2017-January 2018	Non-human: chicken feces	*Escherichia coli*					172/90	
Eltai *et al.*, 2018b [58]	Cross-sectional, February-June 2017	Human: children with lower UTI	*Escherichia coli*					727/201	
Eltai *et al.*, 2018c [59]	Cross-sectional, January 2015-December 2016	Human: stool of healthy food handling workers	*Escherichia coli*					78/456	
Eltai *et al.*, 2020b [60]	Cross-sectional, August 2017 to January 2018	Human: stool of children with acute gastroenteritis	*Escherichia coli* (EPEC and EAEC)	76/33				175/76	
Farag *et al.*, 2016 [61]	Retrospective descriptive, 2004-2012	Human: salmonellosis cases	*Salmonella* spp. (Type B, C1, C2, D, E, and non-classified)	-/94	-/28	-/100	-/47	-/338	
Garcell *et al.*, 2014 [62]	Descriptive, August 2012 to May 2013	Human: health care worker	*Mycobacterium tuberculosis*					202/14	No active TB, but latent TB was detected.
Garcell *et al.*, 2017 [63]	Longitudinal, January 2013 to December 2015	Human: surgical site infection	*Escherichia coli*					603/13	
Ghunaim *et al.*, 2015 [64]	Cross-sectional, 2005-2006	Human: stool of patients with severe diarrhea	*Campylobacter* sp.					26140/497	
			Escherichia coli					26140/732	
			Salmonella sp.					26140/2248	
			Yersinia sp.					26140/732	
Howady *et al.*, 2003 [65]	Retrospective, 1992-1998	Human: spinal TB cases	*Mycobacterium tuberculosis*	-/7		-/25	-/3	-/35	
			Campylobacter spp.					126/9	

Authors and References	Study type and period	Study subjects	Pathogens name	Sample details (Total sample/Total positive)					Additional information
				Qatari	African	South-East Asian	West Asian	Grand Total	
Humphrey *et al.*, 2016 [66]	Prospective, 2015-2016	Human: stool of migrant workers with infectious diarrhea	*Escherichia coli*					126/81	
			Salmonella spp.					128/28	
			Giardia duodenalis					128/12	*Giardia duodenalis* is a synonym of *Giardia lamblia*
Humphrey *et al.*, 2019 [67]	Retrospective cross-sectional, June 2013 to June 2016	Human: Blood donors serum	Chikungunya virus	200/7	977/13	519/46	296/16	1992/82	
Ibrahim *et al.*, 2005a [68]	Retrospective cross-sectional, June 2013 to June 2016	Human: transbronchial biopsy patients	*Mycobacterium tuberculosis*					71/16	
Ibrahim *et al.*, 2009 [69]	Prospective observational, July 2005 to June 2006	Human: serology of Hepatitis cases of Nepali immigrants	Hepatitis E virus					-/86	Hepatitis E is an imported disease in Qatar.
Ibrahim *et al.*, 2005b [70]	Retrospective, January 1996 to December 2002	Human: pulmonary TB	*Mycobacterium tuberculosis*					39/13	Younger people are particularly affected with pleural TB.
Ibrahim *et al.*, 2016 [71]	Cross-sectional, December 2015 to January 2017 .	Human: pulmonary TB	*Mycobacterium tuberculosis*	-/5		-/79	-/21	-/100	
Imam *et al.*, 2015 [72]	Retrospective, January 2006 to December 2012	Human: tuberculous meningitis	*Mycobacterium tuberculosis*	-/2		-/67	-/8	-/80	Tuberculous meningitis is an imported disease. The majority of the tuberculous meningitis patients were originated from the Indian subcontinent, such as Nepal, India, Bangladesh, Pakistan, Sri Lanka.

Authors and References	Study type and period	Study subjects	Pathogens name	Sample details (Total sample/Total positive)					Additional information
				Qatari	African	South-East Asian	West Asian	Grand Total	
Khan *et al*., 2010 [73]	Prospective from July 2007 to June 2008	Human: patients with bacteremia	*Escherichia coli* (ESBL and Non-ESBL)					452/97	
			Salmonella enterica serotype Typhi					452/39	
			Salmonella spp.					452/13	
Khan *et al*., 2012 [74]	Retrospective observational, 2005-2009	Human: peritoneal TB	*Mycobacterium tuberculosis*					54/45	
Khan *et al*., 2013a [75]	A hospital-based retrospective, January 2006-December 2011	Human: adult patient with arthritis	*Escherichia coli*					171/6	
			Mycobacterium tuberculosis					171/3	
Khan *et al*., 2013b [76]	Observational, perspective and case-control; June 2009 – May 2010	Human: pleural TB	*Mycobacterium tuberculosis*					103/72	
Khan *et al*., 2017a [77]	Retrospective, January 2009 to December 2013	Human: meningitis	*Escherichia coli*					110/4	
			Salmonella group B					-/3	
			Listeria monocytogenes					-/1	
Khan, 2017b [78]	Prospective descriptive, January 2004 - January 2005	Human: tuberculous adenitis	*Mycobacterium tuberculosis*	-/5		-/15		-/35	
Minisha *et al*., 2020 [79]	Retrospective, June 2012 to March 2013	Human: UTI of pregnant women	*Escherichia coli*					1588/65	
Mobayedh *et al*., 2002 [80]	Retrospective, July 1997-June 2000	Human: pulmonary TB	*Mycobacterium tuberculosis*					193/58	

Authors and References	Study type and period	Study subjects	Pathogens name	Sample details (Total sample/Total positive)					Additional information
				Qatari	African	South-East Asian	West Asian	Grand Total	
Mohammed *et al.*, 2015 [81]	Cross-sectional	Non-human: Sheep, cattle and camel feces; cattle and camel milk; Cattle udder; sheep, cattle, camel and chicken carcass; Cattle bedding; feed and water trough; chicken abattoir	*Campylobacter coli* *Campylobacter jejuni*					1158/1101	
			Escherichia coli (O157: H7, O26, O45, O103, and O111, STEC)					1158/465	
Nahman and Hammoude, 2003 [82]	Retrospective January 1996 to December 2002	Human: synovial fluid	*Salmonella* sp.					24/1	
Nasrallah *et al.*, 2017 [83]	Cross-sectional June 2013 to June 2016	Human: routine serology of blood of the donors	Hepatitis E virus	1138/133	1138/458	1002/207	1851/319	5854/1263	
Oude Munnink et al., 2020 [84]	Case report	Human	Rabies virus					2	The human cases were imported from Nepal, whereas the animal cases were native Qatari cases
		Fox						1	
		Camel						1	
Royal *et al.*, 2013 [85]	Cross-sectional 2005	Human: Serology among US army personnel	*Coxiella burnetii*					304/26	
Shigidi *et al.*, 2010 [86]	January 2003 to December 2007	Human: peritonitis cases	*Escherichia coli*					241/2	
			Mycobacterium tuberculosis					241/2	

Authors and References	Study type and period	Study subjects	Pathogens name	Sample details (Total sample/Total positive)					Additional information
				Qatari	African	South-East Asian	West Asian	Grand Total	
Szmiegielski *et al*., 1998 [87]	Retrospective, 1987-1995	Human: abdominal TB	*Mycobacterium tuberculosis*					78/59	
Thandassery *et al*., 2014 [88]	Case report	Human: case reports of enteric fever	*Salmonella enterica* serotype Typhi			-/3		-/3	All three cases were imported from Nepal
Thomas *et al*., 2017 [89]	Retrospective, 2008-2015	Human: pleural effusion cases	*Mycobacterium tuberculosis*					401/344	
Thomas, and AlGherbawe, 2014 [90]	Case report	Human: acute myeloid leukemia with pulmonary TB	*Mycobacterium tuberculosis*					-/1	
Uwaydah *et al*., 1991 [91]	Retrospective, 1 October 1989 to 30 September 1990	Human: *Salmonella* bacteremia	*Salmonella enterica* Serotype Typhi	-/3		-/25	-/1	-/100	
Weam *et al*., 2016 [92]	Retrospective, August 2009 to December 2012	Human: stool of gastroenteritis cases	*Campylobacter coli*<br>*Campylobacter fetus*<br>*Campylobacter jejuni*<br>*Campylobacter laridis*<br>*Campylobacter upsaliensis*					423/106	
			Escherichia coli (EPEC 2, EPEC 3, and EPEC 4)					423/208	
			Salmonella spp.<br>(Type B, CI, C2, and D)					423/109	
Zaqout *et al*., 2019 [93]	Case report	Human: Cerebral schistosomiasis	*Schistosoma mansoni*					-/3	All three cases were imported from the Philipines

Authors and References	Study type and period	Study subjects	Pathogens name	Sample details (Total sample/Total positive)					Additional information
				Qatari	African	South-East Asian	West Asian	Grand Total	
Zowawi *et al.*, 2014 [94]	Cross-sectional<br>July 2011-January 2013	Human sample	*Escherichia coli* OXA-48					-/16	

* The authors considered Bangladesh, India, and Sri Lanka from West Asia. We considered these countries as part of South Asia.

Reference

1. Abbas MT, Khan FY, Muhsin SA, Al-Dehwe B, Abukamar M, Elzouki AN. Epidemiology, clinical features and outcome of liver abscess: A single reference center experience in Qatar. Oman Medical Journal. 2014;29(4):260-3. doi: 10.5001/omj.2014.69.
2. Abdelmaguid N, Seleem WS, Soliman AT, Mohamed RS, Elgharbawy FM, Yassin H, et al. Clinical presentations, laboratory analysis and linear growth in 50 neonates and young infants with acute meningitis: One year experience of a single center in Qatar. Mediterranean Journal of Hematology and Infectious Diseases. 2019;11(1). doi: 10.4084/MJHID.2019.028.
3. Abu-Madi M, Behnke JM, Sharma A, Bearden R, Al-Banna N. Prevalence of Virulence/Stress Genes in Campylobacter jejuni from Chicken Meat Sold in Qatari Retail Outlets. PLoS One. 2016;11(6):e0156938. Epub 2016/06/04. doi: 10.1371/journal.pone.0156938. PubMed PMID: 27258021; PubMed Central PMCID: PMCPMC4892673.
4. Abu-Madi M, Boughattas S, Behnke JM, Sharma A, Ismail A. Coproscopy and molecular screening for detection of intestinal protozoa. Parasites & Vectors. 2017;10. doi: 10.1186/s13071-017-2346-7. PubMed PMID: WOS:000410690000005.
5. Abu-Madi MA, Lewis JW, Mikhail M, El-Nagger ME, Behnke JM. Monospecific helminth and arthropod infections in an urban population of brown rats from Doha, Qatar. Journal of Helminthology. 2001;75(4):313-20. doi: 10.1017/s0022149x01000488. PubMed PMID: WOS:000172904500003.
6. Abu-Madi MA, Behnke JM, Mikhail M, Lewis JW, Al-Kaabi ML. Parasite populations in the brown rat Rattus norvegicus from Doha, Qatar between years: the effect of host age, sex and density. Journal of Helminthology. 2005;79(2):105-11. doi: 10.1079/joh2005274. PubMed PMID: WOS:000229679500002.
7. Abu-Madi MA, Al-Ahbabi DA, Al-Mashhadani MM, Al-Ibrahim R, Pal P, Lewis JW. Patterns of parasitic infections in faecal samples from stray cat populations in Qatar. Journal of Helminthology. 2007;81(3):281-6. doi: 10.1017/S0022149X07818505.
8. Abu-Madi MA, Pal P, Al-Thani A, Lewis JW. Descriptive epidemiology of intestinal helminth parasites from stray cat populations in Qatar. J Helminthol. 2008;82(1):59-68. Epub 2008/01/18. doi: 10.1017/s0022149x07870830. PubMed PMID: 18199386.
9. Abu-Madi MA, Behnke JM, Prabhaker KS, Al-Ibrahim R, Lewis JW. Intestinal helminths of feral cat populations from urban and suburban districts of Qatar. Vet Parasitol. 2010;168(3-4):284-92. Epub 2009/12/25. doi: 10.1016/j.vetpar.2009.11.027. PubMed PMID: 20031329.
10. Abu-Madi MA, Behnke JM, Ismail A, Al-Olaqi N, Al-Zaher K, El-Ibrahim R. Comparison of intestinal parasitic infection in newly arrived and resident workers in Qatar. Parasit Vectors. 2011;4:211. Epub 2011/11/08. doi: 10.1186/1756-3305-4-211. PubMed PMID: 22053720; PubMed Central PMCID: PMCPMC3247846.
11. Abu-Madi MA, Behnke JM, Boughattas S, Al-Thani A, Doiphode SH. A decade of intestinal protozoan epidemiology among settled immigrants in Qatar. Bmc Infectious Diseases. 2016;16. doi: 10.1186/s12879-016-1728-3. PubMed PMID: WOS:000380996200001.
12. Abu-Madi MA, Behnke JM, Ismail A, Boughattas S. Assessing the burden of intestinal parasites affecting newly arrived immigrants in Qatar. Parasit Vectors. 2016;9(1):619. Epub 2016/12/03. doi: 10.1186/s13071-016-1906-6. PubMed PMID: 27903290; PubMed Central PMCID: PMCPMC5131437.
13. Abu-Madi MA, Behnke JM, Boughattas S, Al-Thani A, Doiphode SH, Deshmukh A. Helminth infections among long-term-residents and settled immigrants in Qatar in the decade from 2005 to 2014: temporal trends and varying prevalence among subjects from different regional origins. Parasites & Vectors. 2016;9. doi: 10.1186/s13071-016-1433-5. PubMed PMID: WOS:000372438900002.
14. Abu-Madi MA, Al-Molawi N, Behnke JM. Seroprevalence and epidemiological correlates of Toxoplasma gondii infections among patients referred for hospital-based serological testing in Doha, Qatar. Parasit Vectors. 2008;1(1):39. Epub 2008/10/22. doi: 10.1186/1756-3305-1-39. PubMed PMID: 18937857; PubMed Central PMCID: PMCPMC2586624.

15. Abu-Madi MA, Behnke JM, Dabritz HA. Toxoplasma gondii seropositivity and co-infection with TORCH pathogens in high-risk patients from Qatar. Am J Trop Med Hyg. 2010;82(4):626-33. Epub 2010/03/30. doi: 10.4269/ajtmh.2010.09-0530. PubMed PMID: 20348511; PubMed Central PMCID: PMCPMC2844547.
16. Abu-Madi MA, Behnke JM, Doiphode SH. Changing trends in intestinal parasitic infections among long-term-residents and settled immigrants in Qatar. Parasites & Vectors. 2010;3. doi: 10.1186/1756-3305-3-98. PubMed PMID: WOS:000283931300001.
17. Abu-Madi MA, Behnke JM, Doiphode SH. Intestinal parasitic infections among long-term-residents and settled immigrants in Qatar in the period 2005 to 2011. American Journal of Tropical Medicine and Hygiene. 2013;88(6):1185-95. doi: 10.4269/ajtmh.13-0006.
18. Abu-Madi MA, Behnke JM, Ismail A. Patterns of infection with intestinal parasites in Qatar among food handlers and housemaids from different geographical regions of origin. Acta Trop. 2008;106(3):213-20. Epub 2008/05/07. doi: 10.1016/j.actatropica.2008.03.010. PubMed PMID: 18457813.
19. Ahmed MAS, Bansal D, Acharya A, Elmi AA, Hamid JM, Ahmed AMS, et al. Antimicrobial susceptibility and molecular epidemiology of extended-spectrum beta-lactamase-producing Enterobacteriaceae from intensive care units at Hamad Medical Corporation, Qatar. Antimicrobial Resistance and Infection Control. 2016;5. doi: 10.1186/s13756-016-0103-x. PubMed PMID: WOS:000369811900001.
20. Ahmedullah H, Khan FY, Al Maslamani M, Al Soub H, Chacko K, Abu Khattab M, et al. Epidemiological and Clinical Features of Salmonella Typhi Infection Among Adult Patients in Qatar: A Hospital-based Study. Oman Med J. 2018;33(6):468-72. Epub 2018/11/10. doi: 10.5001/omj.2018.87. PubMed PMID: 30410688; PubMed Central PMCID: PMCPMC6206420.
21. Al Alousi FS, Abu Khattab M, Al Soub H, Al-Khal AL, Al-Suwaidi ZD. Value of examining 3 sputum samples in the diagnosis of active pulmonary tuberculosis in qatar. Infectious Diseases in Clinical Practice. 2012;20(3):188-91. doi: 10.1097/IPC.0b013e318245d539.
22. Al Ani AM, Khan FY, Elzouki AN, Al Hajri M, Ibrahim W. Epidemiology of hydatid disease in Qatar: a hospital based study from 2000 to 2013. Asian Pacific Journal of Tropical Medicine. 2014;7:S85-S7. doi: 10.1016/s1995-7645(14)60209-1. PubMed PMID: WOS:000354433300014.
23. Al Jama FE. Risk factors for wound infection after lower segment cesarean section. Qatar Medical Journal. 2012;2012(2):26-31. doi: 10.5339/qmj.2012.2.9.
24. Al Marri MRHA, Al Hail L, Al Otaibi S, Al Marri ND. The time of reactivation of tuberculosis in expatriates in the State of Qatar. Qatar Medical Journal. 2006;15(2):21-3.
25. Al-Marri MR, Almosleh A, Almoslmani Y. Primary tuberculosis of the breast in Qatar: ten year experience and review of the literature. Eur J Surg. 2000;166(9):687-90. Epub 2000/10/18. doi: 10.1080/110241500750008420. PubMed PMID: 11034463.
26. Al Marri MRHA, Al Qatami M, Al Janahi M. The tuberculin skin test in children with tuberculosis in the State of Qatar. Qatar Medical Journal. 2002;11(1):19-22.
27. Al Marri MRHA. The tuberculin skin test in confirmed pulmonary tuberculosis in the state of Qatar: Where we stand? Qatar Medical Journal. 2012;2012(2):16-9. doi: 10.5339/qmj.2012.2.7.
28. Al Soub H, Al Maslamani M, Ahmedullah HS, Shawkat A, Ibrahim FA, Kanbar NA. First case of babesiosis in Qatar: Case report. Jordan Medical Journal. 2016;50(3):161-7. doi: 10.12816/0033487.
29. Al-Aani FK, Abusalah S, Al-Aqeedi R, Ibrahim A. Salmonella meningitis in an adult with type B viral hepatitis and an incidental schwannoma. BMJ Case Rep. 2009;2009. Epub 2009/01/01. doi: 10.1136/bcr.11.2008.1209. PubMed PMID: 21686413; PubMed Central PMCID: PMCPMC3027516.
30. Al-Absi ES, Al-Sadeq DW, Younis MH, Yassine HM, Abdalla OM, Mesleh AG, et al. Performance evaluation of five commercial assays in assessing seroprevalence of HEV antibodies among blood donors. Journal of Medical Microbiology. 2018;67(9):1302-9. doi: 10.1099/jmm.0.000807. PubMed PMID: WOS:000444186500016.
31. Al-Baker ZM, Bodaghi B, Khan SA. Clinical Patterns and Causes of Uveitis in a Referral Eye Clinic in Qatar. Ocular Immunology and Inflammation. 2018;26(2):249-58. doi: 10.1080/09273948.2016.1206573. PubMed PMID: WOS:000426938900015.

32. Al-Dahshan A, Elyamani R, Naja S, Chehab M, Nour M, Elmagboul E, et al. Epidemiological characteristics of a salmonella outbreak among infants in Qatar, 2017. Qatar Med J. 2019;2019(3):12. Epub 2019/12/11. doi: 10.5339/qmj.2019.12. PubMed PMID: 31819858; PubMed Central PMCID: PMCPMC6883746.
33. Alho AM, Lima C, Latrofa MS, Colella V, Ravagnan S, Capelli G, et al. Molecular detection of vector-borne pathogens in dogs and cats from Qatar. Parasites & Vectors. 2017;10. doi: 10.1186/s13071-017-2237-y. PubMed PMID: WOS:000404073800002.
34. Ali M, Shaukat A, Ai-Suwaidi Z, Al-Maslamani M. Tuberculosis of Pancreas, the First Case Reported from Qatar. International Journal of Mycobacteriology. 2019;8(1):101-3. doi: 10.4103/ijmy.ijmy_147_18. PubMed PMID: WOS:000461384800017.
35. Al-Jedah JH, Robinson RK. Nutritional value and microbiological safety of fresh fruit juices sold through retail outlets in Qatar. Pakistan Journal of Nutrition. 2002;1(2):79-81. doi: 10.3923/pjn.2002.79.81.
36. Al-Khal AL, Bener A, Enarson DA. Tuberculosis among garment workers in an Arabian developing country: State of Qatar. Archives of Environmental & Occupational Health. 2005;60(6):295-8. doi: 10.3200/aeoh.60.6.295-298. PubMed PMID: WOS:000245111300004.
37. Al-Marri M. Childhood tuberculosis in the State of Qatar: the effect of a limited expatriate screening programme on the incidence of tuberculosis. International Journal of Tuberculosis and Lung Disease. 2001;5(9):831-7. PubMed PMID: WOS:000170809800007.
38. Al-Marri M. Pattern of mycobacterial resistance to four anti-tuberculosis drugs in pulmonary tuberculosis patients in the State of Qatar after the implementation of DOTS and a limited expatriate screening programme. International Journal of Tuberculosis and Lung Disease. 2001;5(12):1116-21. PubMed PMID: WOS:000172895900007.
39. Al-Marri M, Kirkpatrick MB. Erythrocyte sedimentation rate in childhood tuberculosis: is it still worthwhile? International Journal of Tuberculosis and Lung Disease. 2000;4(3):237-9. PubMed PMID: WOS:000085679900009.
40. Al-Mulla NA, Taj-Aldeen SJ, El Shafie S, Janahi M, Al-Nasser AA, Chandra P. Bacterial bloodstream infections and antimicrobial susceptibility pattern in pediatric hematology/oncology patients after anticancer chemotherapy. Infect Drug Resist. 2014;7:289-99. Epub 2014/11/15. doi: 10.2147/idr.S70486. PubMed PMID: 25395866; PubMed Central PMCID: PMCPMC4226521.
41. Al-Nesf MA, Jerobin J, Al-Alawi AA, El-Kassim M, Mobayed H, Mohammed TRN. Etiology and outcome of hemoptysis in Qatar, a high-resource country with a large expatriate population: A retrospective study. Qatar Medical Journal. 2019;2019(1). doi: 10.5339/qmj.2019.1.
42. Al-Shaer MH, Mansour H, Elewa H, Salameh P, Iqbal F. Treatment outcomes of fixed-dose combination versus separate tablet regimens in pulmonary tuberculosis patients with or without diabetes in Qatar. Bmc Infectious Diseases. 2017;17. doi: 10.1186/s12879-017-2231-1. PubMed PMID: WOS:000397382400003.
43. Al-Shaer MH, Elewa H, Alkabab Y, Nazer LH, Heysell SK. Fixed-dose combination associated with faster time to smear conversion compared to separate tablets of anti-tuberculosis drugs in patients with poorly controlled diabetes and pulmonary tuberculosis in Qatar. Bmc Infectious Diseases. 2018;18. doi: 10.1186/s12879-018-3309-0. PubMed PMID: WOS:000440970600003.
44. Alsoub H, Al Alousi FS. Miliary tuberculosis in Qatar: A review of 32 adult cases. Annals of Saudi Medicine. 2001;21(1-2):16-20. doi: 10.5144/0256-4947.2001.16. PubMed PMID: WOS:000172208300006.
45. Al-Thani A, Baris M, Al-Lawati N, Al-Dhahry S. Characterising the aetiology of severe acute gastroenteritis among patients visiting a hospital in Qatar using real-time polymerase chain reaction. BMC Infectious Diseases. 2013;13(1). doi: 10.1186/1471-2334-13-329.
46. Ben Abid F, Abdel Rahman SASH. A case report of TB versus idiopathic granulomatous mastitis with erythema nodosum, reactive arthritis, cough, and headache. Aging Male. 2018:1-4. Epub 2018/10/09. doi: 10.1080/13685538.2018.1504915. PubMed PMID: 30293476.
47. Boughattas S, Behnke JM, Al-Ansari K, Sharma A, Abu-Alainin W, Al-Thani A, et al. Molecular Analysis of the Enteric Protozoa Associated with Acute Diarrhea in Hospitalized Children. Frontiers in

Cellular and Infection Microbiology. 2017;7. doi: 10.3389/fcimb.2017.00343. PubMed PMID: WOS:000407515800001.
48. Boughattas S, Behnke J, Sharma A, Abu-Madi M. Seroprevalence of Toxoplasma gondii infection in feral cats in Qatar. BMC Vet Res. 2017;13(1):26. Epub 2017/01/20. doi: 10.1186/s12917-017-0952-4. PubMed PMID: 28100230; PubMed Central PMCID: PMCPMC5242050.
49. Boughattas S, Behnke JM, Al-Sadeq D, Ismail A, Abu-Madi M. Cryptosporidium spp., prevalence, molecular characterisation and socio-demographic risk factors among immigrants in Qatar. Plos Neglected Tropical Diseases. 2019;13(10). doi: 10.1371/journal.pntd.0007750. PubMed PMID: WOS:000494982700019.
50. Chang YC, Scaria J, Ibraham M, Doiphode S, Chang Y-F, Sultan A, et al. Distribution and factors associated with Salmonella enterica genotypes in a diverse population of humans and animals in Qatar using multi-locus sequence typing (MLST). Journal of Infection and Public Health. 2016;9(3):315-23. doi: https://doi.org/10.1016/j.jiph.2015.10.013.
51. Derbala M, Elbadri ME, Amer AM, AlKaabi S, Sultan KH, Kamel YM, et al. Aspartate transaminase to platelet ratio index in hepatitis C virus and Schistosomiasis co-infection. World J Gastroenterol. 2015;21(46):13132-9. Epub 2015/12/18. doi: 10.3748/wjg.v21.i46.13132. PubMed PMID: 26674154; PubMed Central PMCID: PMCPMC4674732.
52. Dousa KM, Hamad A, Albirair M, Al Soub H, Elzouki AN, Alwakeel MI, et al. Impact of Diabetes Mellitus on the Presentation and Response to Treatment of Adults With Pulmonary Tuberculosis in Qatar. Open Forum Infectious Diseases. 2019;6(1). doi: 10.1093/ofid/ofy335. PubMed PMID: WOS:000460636900014.
53. Dubey JP, Pas A, Rajendran C, Kwok OCH, Ferreira LR, Martins J, et al. Toxoplasmosis in Sand cats (Felis margarita) and other animals in the Breeding Centre for Endangered Arabian Wildlife in the United Arab Emirates and Al Wabra Wildlife Preservation, the State of Qatar. Veterinary Parasitology. 2010;172(3):195-203. doi: https://doi.org/10.1016/j.vetpar.2010.05.013.
54. Ehlayel MS, Bener A, Laban MA. Primary immunodeficiency diseases in children: 15 year experience in a tertiary care medical center in Qatar. J Clin Immunol. 2013;33(2):317-24. Epub 2012/10/12. doi: 10.1007/s10875-012-9812-y. PubMed PMID: 23054346.
55. El-Nemr IM, Mushtaha M, Sundararaju S, Fontejon C, Suleiman M, Tang P, et al. Application of MALDI Biotyper System for Rapid Identification of Bacteria Isolated from a Fresh Produce Market. Current Microbiology. 2019;76(3):290-6. doi: 10.1007/s00284-018-01624-1.
56. Eltai N, Al Thani AA, Al-Hadidi SH, Abdfarag EA, Al-Romaihi H, Mahmoud MH, et al. Antibiotic resistance profile of commensal Escherichia coli isolated from healthy sheep in Qatar. J Infect Dev Ctries. 2020;14(2):138-45. Epub 2020/03/09. doi: 10.3855/jidc.11827. PubMed PMID: 32146447.
57. Eltai NO, Abdfarag EA, Al-Romaihi H, Wehedy E, Mahmoud MH, Alawad OK, et al. Antibiotic Resistance Profile of Commensal Escherichia coli Isolated from Broiler Chickens in Qatar. J Food Prot. 2018;81(2):302-7. Epub 2018/01/26. doi: 10.4315/0362-028x.Jfp-17-191. PubMed PMID: 29369690.
58. Eltai NO, Al Thani AA, Al-Ansari K, Deshmukh AS, Wehedy E, Al-Hadidi SH, et al. Molecular characterization of extended spectrum beta -lactamases enterobacteriaceae causing lower urinary tract infection among pediatric population. Antimicrobial Resistance and Infection Control. 2018;7. doi: 10.1186/s13756-018-0381-6. PubMed PMID: WOS:000440667900002.
59. Eltai NO, Yassine HM, Al Thani AA, Abu Madi MA, Ismail A, Ibrahim E, et al. Prevalence of antibiotic resistant Escherichia coli isolates from fecal samples of food handlers in Qatar. Antimicrob Resist Infect Control. 2018;7:78. Epub 2018/07/10. doi: 10.1186/s13756-018-0369-2. PubMed PMID: 29983931; PubMed Central PMCID: PMCPMC6019201.
60. Eltai NO, Al Thani AA, Al Hadidi SH, Al Ansari K, Yassine HM. Antibiotic resistance and virulence patterns of pathogenic Escherichia coli strains associated with acute gastroenteritis among children in Qatar. BMC Microbiol. 2020;20(1):54. Epub 2020/03/08. doi: 10.1186/s12866-020-01732-8. PubMed PMID: 32143566.
61. Farag E, Garcell HG, Ganesan N, Ahmed SN, Al-Hajri M, Al Thani SM, et al. A retrospective epidemiological study on the incidence of salmonellosis in the State of Qatar during 2004-2012. Qatar Med

J. 2016;2016(1):3. Epub 2016/09/16. doi: 10.5339/qmj.2016.3. PubMed PMID: 27630822; PubMed Central PMCID: PMCPMC5009826.
62. Garcell HG, Ramirez EC, Contreras AK, Garcia FG. Latent tuberculosis infection in healthcare workers at a community hospital in Qatar. Journal of Infection and Public Health. 2014;7(4):356-9. doi: 10.1016/j.jiph.2014.02.001. PubMed PMID: WOS:000354963100013.
63. Garcell HG, Arias AV, Sandoval CA, Garcia EG, Gamboa ME, Sado AB, et al. Incidence and Etiology of Surgical Site Infections in Appendectomies: A 3-Year Prospective Study. Oman Med J. 2017;32(1):31-5. Epub 2017/01/04. doi: 10.5001/omj.2017.06. PubMed PMID: 28042400; PubMed Central PMCID: PMCPMC5187404.
64. Ghunaim H, Behnke JM, Aigha I, Sharma A, Doiphode SH, Deshmukh A, et al. Analysis of resistance to antimicrobials and presence of virulence/stress response genes in Campylobacter isolates from patients with severe diarrhoea. PLoS ONE. 2015;10(3). doi: 10.1371/journal.pone.0119268.
65. Howady FS, Al Soub H, Al Khal AL. Spinal tuberculosis in Qatar. Qatar Medical Journal. 2003;12(2):85-8.
66. Humphrey JM, Ranbhise S, Ibrahim E, Al-Romaihi HE, Farag E, Abu-Raddad LJ, et al. Multiplex Polymerase Chain Reaction for Detection of Gastrointestinal Pathogens in Migrant Workers in Qatar. Am J Trop Med Hyg. 2016;95(6):1330-7. Epub 2016/12/09. doi: 10.4269/ajtmh.16-0464. PubMed PMID: 27928081; PubMed Central PMCID: PMCPMC5154446.
67. Humphrey JM, Al-Absi ES, Hamdan MM, Okasha SS, Al-Trmanini DM, El-Dous HG, et al. Dengue and chikungunya seroprevalence among Qatari nationals and immigrants residing in Qatar. PLoS One. 2019;14(1):e0211574. Epub 2019/02/01. doi: 10.1371/journal.pone.0211574. PubMed PMID: 30703150; PubMed Central PMCID: PMCPMC6355019.
68. Ibrahim AS, Allangawi MH, Sattar HA, Mobyed HS, Almohammed AA. Indications, diagnostic yields and complications of transbronchial biopsy over 5 years in the State of Qatar. Saudi Medical Journal. 2005;26(4):641-5. PubMed PMID: WOS:000229422200025.
69. Ibrahim AS, Alkhal A, Jacob J, Ghadban W, Almarri A. Hepatitis E in Qatar imported by expatriate workers from Nepal: epidemiological characteristics and clinical manifestations. J Med Virol. 2009;81(6):1047-51. Epub 2009/04/22. doi: 10.1002/jmv.21474. PubMed PMID: 19382273.
70. Ibrahim WH, Ghadban W, Khinji A, Yasin R, Soub H, Al-Khal AL, et al. Does pleural tuberculosis disease pattern differ among developed and developing countries. Respiratory Medicine. 2005;99(8):1038-45. doi: 10.1016/j.rmed.2004.12.012.
71. Ibrahim WH, Alousi FH, Al-Khal A, Bener A, AlSalman A, Aamer A, et al. Diagnostic Delay among Adults with Pulmonary Tuberculosis in a High Gross Domestic Product Per Capita Country: Reasons and Magnitude of the Problem. International Journal of Preventive Medicine. 2016;7. doi: 10.4103/2008-7802.193091. PubMed PMID: WOS:000399002500003.
72. Imam YZB, Ahmedullah HS, Akhtar N, Chacko KC, Kamran S, Al Alousi F, et al. Adult Tuberculous Meningitis in Qatar: A Descriptive Retrospective Study from its Referral Center. European Neurology. 2015;73(1-2):90-7. doi: 10.1159/000368894. PubMed PMID: WOS:000348410900017.
73. Khan FY, Elshafie SS, Almaslamani M, Abu-Khattab M, El Hiday AH, Errayes M, et al. Epidemiology of bacteraemia in Hamad general hospital, Qatar: a one year hospital-based study. Travel Med Infect Dis. 2010;8(6):377-87. Epub 2010/11/16. doi: 10.1016/j.tmaid.2010.10.004. PubMed PMID: 21074495.
74. Khan FY, Al-Muzrakchi AM, Elbedawi MM, Al-Muzrakchi AA, Al Tabeb A. Peritoneal tuberculosis in Qatar: A five-year hospital-based study from 2005 to 2009. Travel Medicine and Infectious Disease. 2012;10(1):25-31. doi: 10.1016/j.tmaid.2011.12.001. PubMed PMID: WOS:000301879300003.
75. Khan FY, Abu-Khattab M, Baagar K, Mohamed SF, Elgendy I, Anand D, et al. Characteristics of patients with definite septic arthritis at Hamad General Hospital, Qatar: A hospital-based study from 2006 to 2011. Clinical Rheumatology. 2013;32(7):969-73. doi: 10.1007/s10067-013-2211-9.
76. Khan FY, Hamza M, Omran AH, Saleh M, Lingawi M, Alnaqdy A, et al. Diagnostic value of pleural fluid interferon-gamma and adenosine deaminase in patients with pleural tuberculosis in Qatar. International Journal of General Medicine. 2013;6:13-8. doi: 10.2147/IJGM.S39345.

77. Khan FY, Abu-Khattab M, Almaslamani EA, Hassan AA, Mohamed SF, Elbuzdi AA, et al. Acute Bacterial Meningitis in Qatar: A Hospital-Based Study from 2009 to 2013. BioMed Research International. 2017;2017. doi: 10.1155/2017/2975610.
78. Khan FY. Clinical pattern of tuberculous adenitis in Qatar: Experience with 35 patients. Scandinavian Journal of Infectious Diseases. 2009;41(2):128-34. doi: 10.1080/00365540802578991. PubMed PMID: WOS:000262417400009.
79. Minisha F, Mohamed M, Abdulmunem D, El Awad S, Zidan M, Abreo M, et al. Bacteriuria in pregnancy varies with the ambiance: a retrospective observational study at a tertiary hospital in Doha, Qatar. J Perinat Med. 2019;48(1):46-52. Epub 2019/10/31. doi: 10.1515/jpm-2018-0419. PubMed PMID: 31665122.
80. Mobayedh HMS, Sattar HA, Ghadban WK, Al Khal AL, Al Soub H, Al Alousi FS, et al. Diagnostic value of fiberoptic bronchoscopy in suspected pulmonary tuberculosis in the state of Qatar. Qatar Medical Journal. 2002;11(2):39-42.
81. Mohammed HO, Stipetic K, Salem A, McDonough P, Chang YF, Sultan A. Risk of Escherichia coli O157:H7, Non-O157 Shiga Toxin-Producing Escherichia coli, and Campylobacter spp. in Food Animals and Their Products in Qatar. J Food Prot. 2015;78(10):1812-8. Epub 2015/09/27. doi: 10.4315/0362-028x.Jfp-14-596. PubMed PMID: 26408129.
82. Nahman A, Hammoudeh M. Pyogenic arthritis in Qatar. Qatar Medical Journal. 2003;12(1):25-7.
83. Nasrallah GK, Al Absi ES, Ghandour R, Ali NH, Taleb S, Hedaya L, et al. Seroprevalence of hepatitis E virus among blood donors in Qatar (2013-2016). Transfusion. 2017;57(7):1801-7. Epub 2017/04/30. doi: 10.1111/trf.14116. PubMed PMID: 28453178.
84. Oude Munnink BB, Farag EABA, GeurtsvanKessel C, Schapendonk C, van der Linden A, Kohl R, et al. First molecular analysis of rabies virus in Qatar and clinical cases imported into Qatar, a case report. International Journal of Infectious Diseases. 2020;96:323-6. doi: https://doi.org/10.1016/j.ijid.2020.04.070.
85. Royal J, Riddle MS, Mohareb E, Monteville MR, Porter CK, Faix DJ. Seroepidemiologic Survey for Coxiella burnetii Among US Military Personnel Deployed to Southwest and Central Asia in 2005. American Journal of Tropical Medicine and Hygiene. 2013;89(5):991-5. doi: 10.4269/ajtmh.12-0174. PubMed PMID: WOS:000326904100030.
86. Shigidi MM, Fituri OM, Chandy SK, Asim M, Al Malki HA, Rashed AH. Microbial spectrum and outcome of peritoneal dialysis related peritonitis in Qatar. Saudi J Kidney Dis Transpl. 2010;21(1):168-73. Epub 2010/01/12. PubMed PMID: 20061719.
87. Szmiegielski W, Venkataraman B, Ejeckam GC, Jarikre LN. Abdominal tuberculosis in Qatar: a clinico-radiological study. International Journal of Tuberculosis and Lung Disease. 1998;2(7):563-8. PubMed PMID: WOS:000074539600007.
88. Thandassery RB, Sharma M, Abdelmola A, Derbala MFM, Al Kaabi SR. Uncommon gastrointestinal complications of enteric fever in a non-endemic country. Qatar Medical Journal. 2014;2014(1). doi: 10.5339/qmj.2014.7.
89. Thomas M, Ibrahim WH, Raza T, Mushtaq K, Arshad A, Ahmed M, et al. Medical thoracoscopy for exudative pleural effusion: an eight-year experience from a country with a young population. Bmc Pulmonary Medicine. 2017;17. doi: 10.1186/s12890-017-0499-y. PubMed PMID: WOS:000416046600002.
90. Thomas M, AlGherbawe M. Acute myeloid leukemia presenting with pulmonary tuberculosis. Case Rep Infect Dis. 2014;2014:865909. Epub 2014/07/06. doi: 10.1155/2014/865909. PubMed PMID: 24987539; PubMed Central PMCID: PMCPMC4058510.
91. Uwaydah AK, Matar I, Chacko KC, Davidson JC. The emergence of antimicrobial resistant Salmonella typhi in Qatar: epidemiology and therapeutic implications. Transactions of the Royal Society of Tropical Medicine and Hygiene. 1991;85(6):790-2. doi: https://doi.org/10.1016/0035-9203(91)90457-A.
92. Weam B, Abraham M, Doiphode S, Peters K, Ibrahim E, Sultan A, et al. Foodborne Bacterial Pathogens Associated with the Risk of Gastroenteritis in the State of Qatar. Int J Health Sci (Qassim).

2016;10(2):197-207. Epub 2016/04/23. PubMed PMID: 27103902; PubMed Central PMCID: PMCPMC4825893.
93. Zaqout A, Abid FB, Murshed K, Al-Bozom I, Al-Rumaihi G, Al Soub H, et al. Cerebral schistosomiasis: Case series from Qatar. International Journal of Infectious Diseases. 2019;86:167-70. doi: https://doi.org/10.1016/j.ijid.2019.07.002.
94. Zowawi HM, Sartor AL, Balkhy HH, Walsh TR, Al Johani SM, AlJindan RY, et al. Molecular characterization of carbapenemase-producing Escherichia coli and Klebsiella pneumoniae in the countries of the Gulf cooperation council: dominance of OXA-48 and NDM producers. Antimicrob Agents Chemother. 2014;58(6):3085-90. Epub 2014/03/19. doi: 10.1128/aac.02050-13. PubMed PMID: 24637692; PubMed Central PMCID: PMCPMC4068443.
